# Impact of gap anisotropy of Polar and Anderson-Brinkman-Morel p-wave superconductors on thermoelectric properties of quantum dot hybrids

**DOI:** 10.1038/s41598-026-46160-2

**Published:** 2026-04-16

**Authors:** Vrishali Sonar, Piotr Trocha

**Affiliations:** Institute of Spintronics and Quantum Information, Faculty of Physics and Astronomy, Adam Mickiewicz University, Poznań, 61-614 Poland

**Keywords:** Gap anisotropy modeling, Triplet superconductor, Symmetry-orientation based transport, Triplet Andreev reflection, Keldysh Green’s functions, Materials science, Physics

## Abstract

We theoretically investigate the thermoelectric transport properties of a hybrid device consisting of a quantum dot (QD) coupled to a ferromagnetic lead and a *p*-wave, spin-triplet superconducting electrode. We focus on two distinct phases - the Polar and Anderson-Brinkman-Morel (ABM) - both having anisotropic gap structure and pure spin-triplet pairing. To capture the momentum-dependent tunneling between QD and the triplet superconductor (TSC), we introduce a phenomenological angle-dependent weighting of the QD-TSC coupling and analyze two configurations in which the superconducting symmetry axis is parallel or perpendicular to the tunneling axis. Employing the Keldysh Green’s function formalism in the linear response regime, we compute key transport coefficients - electrical and thermal conductance, thermopower, and the thermoelectric figure of merit - based on the anisotropy strength parameter, which is the central focus of this work rather than strong intra-dot correlation physics. Transport coefficients exhibit phase, geometry and anisotropy strength-sensitive behaviors, thereby making them potential probes of superconducting order parameter and nodal orientation. We demonstrate that neglecting anisotropy in modeling conceals important qualitative signatures. Our formulation allows to separately quantify the contribution of triplet Andreev reflection and quasiparticle tunneling and shows that, by mere rotation of the crystallographic axis of the superconductor, it is possible to obstruct or maximize the effective (triplet) Andreev reflection. In the ABM state, the origin of orientation-dependent suppression of Andreev reflection is traced to the azimuthal phase dependence. Moreover, the thermal conductance is enhanced by a few orders of magnitude compared to the conventional *s*-wave case. The results demonstrate that the anisotropic, orientation-dependent triplet gap strongly governs transport, offering experimentally accessible signatures of the superconducting phase and nodal structure.

## Introduction

*P*-wave triplet superconductors form a subclass within the broader category of *unconventional superconductors*, characterized by *Cooper pairs* with total spin $S = 1$. To satisfy the antisymmetry requirement of the fermionic wavefunction under particle exchange, the spin-singlet state ($S = 0$) must be paired with a symmetric orbital wavefunction. Conversely, the symmetric spin-triplet state ($S = 1$) requires an odd-parity orbital wavefunction. As a result, spin-singlet superconductors exhibit even-parity spatial functions, such as $L = 0$ (*s*-wave) and $L = 2$ (*d*-wave), while spin-triplet states possess odd-parity spatial functions, such as $L = 1$ (*p*-wave), $L = 3$ (*f*-wave), and so on. In the crystal, the quantum numbers S and L are not strictly well defined, but the terms *s*-, *p*-, and *d*-wave remain convenient descriptors of the symmetry of the order parameter^1,2^. The Cooper pair wavefunction in a *p*-wave superconductor carries angular momentum, and these systems are particularly intriguing due to their spin-triplet pairing and the anisotropic nature of the gap structure, which features nodal points where the excitation energy gap vanishes. The first example of *p*-wave superconductivity (or superfluidity, to be precise) was observed in ${}^{3}{He}$ and remains a quintessential model for this state of matter^3,4^.

Notable *p*-wave triplet phases include the Anderson-Brinkman-Morel (ABM or chiral *p*-wave) state,^5,6^ the Balian Werthamer (BW) state,^7^ and the Polar state^8^. A classic solid-state candidate has been $Sr_{2}RuO_{4}$^9–11^, long regarded as a spin-triplet, chiral superconductor. Although recent experiments have defied the original $p_x+ip_y$^12,13^ pairing symmetry, it remains one of the most deeply studied unconventional superconductors, giving an understanding of how correlations, crystal symmetry, and spin-orbit coupling can result in non-*s*-wave pairing. $UTe_2$ has recently emerged as a prominent heavy-fermion superconductor^14–16^, exhibiting multiple phases, unusually high critical fields, and signatures of non-unitary *p*-wave pairing^17^, making it a key platform for studying topological and spin-triplet superconductivity and potential Majorana excitations. Although the exact nature of its superconducting phases remains under active debate.

*P*-wave superconductors have attracted great attention for being endowed with rich quantum phenomena. In the bulk, the odd-parity and anisotropic gap structure^8,18^ give rise to nontrivial topological character. These materials support protected zero-energy boundary states that directly link *p*-wave superconductivity to Majorana physics. Therefore, the prospect of realizing Majorana zero modes (MZMs) has become a major motivation for studying *p*-wave superconductors^19–23^.

Beyond topological factors, some *p*-wave superconducting phases possess chirality, which is a preferred orbital rotation of Cooper pairs in momentum space due to the complex phase structure of the order parameter. Chirality modifies the relative phases between electron and hole amplitudes, hence influencing Andreev reflection and quasiparticle tunneling at hybrid interfaces. As a result, chiral superconductors can display direction-dependent conductance and thermoelectric responses, as observed in recent theoretical^24–29^ and experimental transport measurements^27,30–32^.

Additionally, chirality or non-unitarity^33^ in the order parameter can break time-reversal symmetry (TRS), leading to distinctive spectral features, spin response, and transport. Such TRS-breaking has been the focus of extensive theoretical and experimental research^34–38^. The spin-triplet pairing enables the transport of spin-polarized Cooper pairs^39–44^, allowing dissipationless, pure spin currents without net charge flow. This makes *p*-wave superconductors particularly promising for superconducting spintronics, where spin manipulation and spin-based information transfer are desired. The inherent anisotropy of *p*-wave strongly influences quasiparticle scattering and energy transport. Particularly, the thermal conductance becomes highly sensitive to the momentum dependence and orientation of the gap function, as variations in the nodal structure significantly alter the low-energy quasiparticle density distribution^45–50^. These features make spin-triplet superconductors highly attractive for energy-efficient spin-caloritronics, quantum technologies, and spintronics. Despite these appealing properties, identifying *p*-wave superconductivity remains challenging. Many proposed states exhibit thermodynamic responses that closely resemble the *s*-wave or *d*-wave order, making it difficult to clearly identify phase and pairing symmetry^51,52^. Candidate materials are also highly sensitive to temperature, pressure, and disorder, requiring high-quality single crystals for reliable measurements^53,54^. Moreover, they often exhibit multiple closely competing superconducting sub-phases, and the microscopic origin of the pairing correlations is not fully clear^55^. Given these challenges, theoretical modeling can play a crucial role in elucidating the transport properties of unconventional *p*-wave superconductors.

In contrast, hybrid systems incorporating *p*-wave remain relatively unexplored. Only a limited number of theoretical or numerical studies have addressed these systems^56–59^. Some recent works have analyzed electrical conductance in junctions with *d*-wave-like superconducting leads^60^, modeling point-contact Andreev reflection (PCAR) experiments for prospective ABM-state material^61^ and the proposition of measurable signatures of current-carrying triplet Cooper pairs^40,62^. Despite these efforts, the impact of anisotropic and triplet pairing on observables in hybrid systems has a large scope to be studied, thus motivating theoretical work to assist experiments and clarify definitive signatures of *p*-wave phase.

In this work, we study a hybrid device consisting of a quantum dot coupled to a ferromagnetic (or metallic) lead and a *p*-wave superconducting terminal (TSC). Without assuming a specific material, we focus on two characteristic phases - the Polar state and the Anderson-Brinkman-Morel (ABM) state. We formulate a phenomenological model to incorporate an anisotropic gap function into the QD-TSC coupling. We compute linear-response transport properties, viz., electrical conductance, thermopower, thermal conductance, and the thermoelectric figure of merit. The article is organized as follows, section Theoretical Description introduces the Hamiltonian, Green’s function, the linear-response formalism, and modeling of anisotropic QD-TSC coupling. Section Numerical Results and Discussion presents benchmark results for the Polar state without weighting, including a comparison with the conventional *s*-wave case. Section Effect of Gap Anisotropy on Transport Properties contains the central results of this article, i.e. thermoelectric coefficients for the Polar and ABM states under weighting in both parallel and perpendicular configurations. Section Summary summarizes the main outcomes. The supplementary information includes the analytical form of Green’s function and unweighted self-energy; furthermore, it includes figures of tunneling coefficients and weighted self-energy.

## Theoretical description

### Model

**Figure 1:**
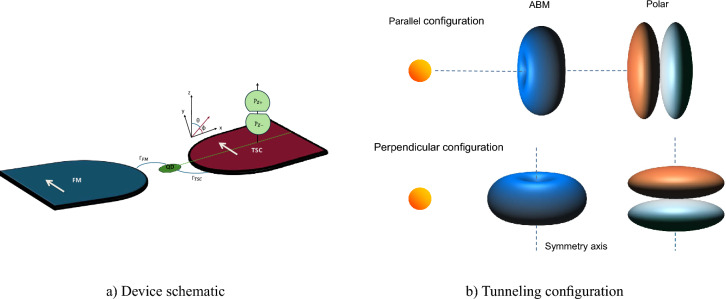
(**a**) Schematic of the model device. The device setup consists of a quantum dot coupled to two bulk leads. One lead is ferromagnet (FM) with spin polarization *p*, and the other is a triplet superconductor (TSC), considered in either a Polar or ABM state. The white arrow in FM lead indicates the direction of magnetization (also assumed to be the $\hat{z}$ direction in the *spin space*) of the FM lead. The arrow in TSC lead indicates the $\uparrow$ spin quantization direction. For magnetization-direction-independent transport, the two arrows need to be parallel. (**b**) Tunneling scheme for parallel and perpendicular configurations showing the 3D anisotropic gap functions for Polar and ABM state. The superconducting gap structure shown in the figure is in ‘momentum space’ of the electron, whereas the spin space can generally have an independent orientation.

The hybrid system consisting of metallic (ferromagnetic) lead and an unconventional (triplet) superconductor coupled to a single energy level quantum dot, as shown in Fig. 1, can be modeled using the Anderson impurity model and the generalized BCS theory. The effective Hamiltonian for the device is given by

1 $$\begin{aligned} H=H_{FM}+H_{QD}+H_{TSC}+H_{T}. \end{aligned}$$

Here $H_{FM}$ represents Hamiltonian of the ferromagnetic lead, describing non-interacting electrons $H_{FM}= \sum _{k\sigma }^{}\varepsilon _{ k\sigma }c_{k\sigma }^{\dagger }c_{k\sigma }^{}$. The spin dependent dispersion of the lead $\varepsilon _{k\sigma }$ in the mean-field Stoner form^63,64^, assuming magnetization is aligned along $\hat{z}$ (in spin space of FM) direction is given as,

2 $$\begin{aligned} \varepsilon _{k\sigma } = \frac{\hbar ^2 k^2}{2m^*} -\mu -\sigma h_z, \end{aligned}$$

where, $\frac{\hbar ^2k^2}{2m^*}$ is the bare kinetic energy, $\mu$ is the chemical potential, ${h_z}$ is the exchange field, giving rise to exchange splitting of 2h_z_, describing magnetization of the ferromagnet. We set $\sigma = +1$ for spin-up ($\uparrow$) and $\sigma = -1$ for spin-down ($\downarrow$).

Further, $H_{QD}=\sum _{\sigma }^{} \varepsilon _{d}^{}d_{\sigma }^{\dagger }d_{\sigma }^{}$ represents the non interacting quantum dot with a spin-independent single energy level $\varepsilon _d$. The present work focuses on the effects of anisotropic triplet superconducting correlations on transport rather than strong intra-dot correlation effects^65–68^. Therefore, the quantum dot is modeled as a non-interacting resonant level.

$H_{TSC}$ denotes Hamiltonian of unconventional superconductor including triplet pairing^18,69^

3 $$\begin{aligned} H_{TSC}=\sum _{k,\sigma } \varepsilon _{k}^{}b_{k\sigma }^{\dagger }b_{k\sigma }^{} +\frac{1}{2}\sum _{k} \sum _{\sigma _{1},\sigma _{2}}^{}(\Delta _{\sigma _{1}\sigma _{2}}^kb_{k\sigma _{1} }^{\dagger }b_{{-k}\sigma _{2}}^{\dagger }+\Delta _{\sigma _{1}\sigma _{2}}^{k*}b_{-k\sigma _{2} }^{}b_{k\sigma _{1}}^{}). \end{aligned}$$

Here $\sigma _1, \sigma _2 = \uparrow , \downarrow$, thus Eq. 3 indicates that all the permutations of electron’s spin pairing are possible in *Cooper pairs*. Next, $b_{k\sigma _{1}}$ is the single electron annihilation operator of an electron of momentum *k* and spin $\sigma _1$ in the superconductor. Notice the explicit dependence of $\Delta _{\sigma _1\sigma _2}^k$ on the wave vector k. Unlike the BCS treatment for conventional *s-wave* superconducting state, which has an isotropic gap parameter, independent of the direction and magnitude of the momentum of electrons in the Cooper pair. In general, the superconducting gap parameter for a triplet superconductor is momentum dependent . Here we do not attribute the cause of attractive potential to a specific type of interaction, as is the electron-phonon interaction to the *s*-wave. The strength of this attractive potential is implicit in the gap parameter $\Delta ^k$. The gap parameter for the unconventional superconductor can be defined in the matrix form as^18^,

4 $$\begin{aligned} \Delta ({k}) = \begin{bmatrix} \Delta _{\uparrow \uparrow }^{{k}} & \Delta _{\uparrow \downarrow }^{{k}} \\ \Delta _{\uparrow \downarrow }^{{k}} & \Delta _{\downarrow \downarrow }^{{k}} \end{bmatrix}. \end{aligned}$$

Finally, $H_{T}$ stands for Hamiltonian for the tunneling between QD and the leads, and is given as;

5 $$\begin{aligned} H_{T}=\sum _{k\sigma }^{} V_{k\sigma }^{FM}c_{k\sigma }^{\dagger }d_{\sigma }^{} +\sum _{k\sigma }^{} V_{k\sigma }^{TSC}b_{k\sigma }^{\dagger }d_{\sigma }^{} + \mathrm{H.C.}\end{aligned}$$

Here, $V_{k\sigma }^{FM}$, $V_{k\sigma }^{TSC}$ are spin-conserving elements of the tunneling matrix, indicating the amplitude of tunneling between quantum dot and the leads for $\sigma =\uparrow , \downarrow$.

### Green’s function formalism

According to our definition of the Hamiltonian in Eq. 1, the current flowing through the device is always mediated by the quantum dot. This current can be formulated using the Green function technique. To achieve this, we first introduce a four-component Nambu spinor that describes the particle-hole space of the quantum dot as follows,

6 $$\begin{aligned} \boldsymbol{\Psi }=\begin{pmatrix} d_\uparrow ^\dagger & d_\downarrow & d_\downarrow ^\dagger & d_\uparrow \\ \end{pmatrix}. \end{aligned}$$

With the help of Eq. (6), the retarded Green’s function in time domain is defined as

7 $$\begin{aligned} \boldsymbol{\rm G}^r= -i\theta (t-t') \left\langle [{\boldsymbol{\Psi }(t)}^{\dagger }|\boldsymbol{\Psi }(t')] \right\rangle . \end{aligned}$$

The bold symbols in equations indicate a matrix throughout the article. A detailed formulation of obtaining the retarded Green’s function $\boldsymbol{\rm G}^r$ by employing the equation of motion (EOM) method is provided in the supplementary information. The advanced Green’s function is given as $\boldsymbol{\rm G}^a= {\boldsymbol{\rm G}}^{r\dagger }$. Further, it is the lesser Green function $\boldsymbol{\rm G}^{<}$ that is directly related to the kinetic properties of the system, such as the charge or the heat current, and it is determined by the Keldysh relation;

8 $$\begin{aligned}\mathbf{G}^{<}= \mathbf{G}^{r}\mathbf {\Sigma }^{<}\mathbf{G}^{a} \end{aligned}$$

where the total lesser self-energy matrix is given by,

9 $$\begin{aligned} \mathbf {\Sigma }^{<} = \mathbf {\Sigma _{FM}}^{<}+\mathbf {\Sigma _{TSC}}^{<}. \end{aligned}$$

In the non-interacting case, the lesser self-energy is related to the retarded $\boldsymbol{\Sigma }^r$ and advanced self-energy $\boldsymbol{\Sigma }^a (= {\boldsymbol{\Sigma }^r}^{\dagger })$ as $\boldsymbol{\Sigma }_{i}^{<} = (\boldsymbol{\Sigma }^{a}-\boldsymbol{\Sigma }^{r})\boldsymbol{\rm F}_{i}$ for $i=FM, TSC$. Here, $\boldsymbol{\rm F}_{i}$ is a matrix of the Fermi-Dirac distribution function of the leads, $\mathbf{F}_{FM} = diag(f(\varepsilon -\mu _{FM}), \ f(\varepsilon +\mu _{FM}), \ f(\varepsilon -\mu _{FM}), \ f(\varepsilon +\mu _{FM}))$, and $\mathbf{F}_{TSC} = diag(f(\varepsilon -\mu _{TSC}), \ f(\varepsilon -\mu _{TSC}), \ f(\varepsilon -\mu _{TSC}), \ f(\varepsilon -\mu _{TSC}))$, $\mu _{FM, TSC}$ is the chemical potential of respective lead. The total retarded self-energy of the system is $\boldsymbol{\Sigma }^r= \boldsymbol{\Sigma }_{FM}^r+\boldsymbol{\Sigma }_{TSC}^r$. For simplicity, we assume that the coupling amplitudes for tunneling between the QD and the leads, $V_{k\sigma }^{FM}, V_{k\sigma }^{TSC}$ in Eq. 5 are independent of the spin and all spin dependence is captured by the relevant spin-dependent density of states in a given lead. Thus, the spin-dependent tunneling coupling strength is introduced as $\Gamma _{i\sigma }=2\pi \langle {|V_{i}|}^{2} \rangle \rho _{i\sigma }$, where $i=FM, TSC$, and $\langle ..\rangle$ indicates the average over *k* , and $\rho _{i\sigma }$ denotes the density of states of the respective lead. The $\rho _{i\sigma }$ for a superconductor is taken in its normal state. The spin-dependent density of states for the ferromagnetic lead can be parametrized as $\rho _{FM \uparrow (\downarrow )} = \rho _{FM}(1\pm p)$, where $\rho _{FM}$ is the total electron density in the FM lead taken in the wide-band limit and *p* is spin- polarization factor. The resulting coupling matrix $\boldsymbol{\Gamma }_{FM} = diag({\Gamma _{\uparrow }, \Gamma _{\downarrow }, \Gamma _{\downarrow }, \Gamma _{\uparrow }})$ with $\Gamma _{{FM},\uparrow (\downarrow )}=\Gamma _{FM}(1\pm p)$. Accordingly, the self-energy $\boldsymbol{\Sigma }^r_{FM}$ takes the form

10 $$\begin{aligned} \boldsymbol{\Sigma }^r_{FM} = -\frac{i}{2} \boldsymbol{\Gamma }_{FM}. \end{aligned}$$

For conventional *s*-wave superconductor, the quasiparticle density of states has a form,

11 $$\begin{aligned} \rho _{s-wave}=\theta (|\varepsilon |-\Delta )\frac{|\varepsilon |}{\sqrt{\varepsilon ^2 -\Delta ^2}} \end{aligned}$$

in which, the gap function $\Delta$ is isotropic. However, the self-energy associated with the triplet superconductor lead is determined by the particular form of the gap function characterizing the phase, as well as by the orientation of the gap’s symmetry axis relative to the primary tunneling direction, both of which are rooted in the anisotropic nature of the superconducting gap. We further characterize the gap structure, density of states, and associated self-energy for Polar and ABM superconducting phases.

#### Polar and ABM state gap functions

Both the phases exhibit an anisotropic gap structure, therefore, let ${\hat{k}}$ to indicate the direction of the momentum of electrons involved in triplet superconducting pairing, then following Eq. 4, the gap function can be written in the matrix form^8^

12 $$\begin{aligned} \Delta (k)_{Pol} = \Delta _{0} \begin{bmatrix} {\hat{k}_{z}} & 0 \\ 0 & {\hat{k}_{z}} \\ \end{bmatrix} =\Delta _{0} \begin{bmatrix} \cos {\theta } & 0 \\ 0 & \cos {\theta } \\ \end{bmatrix} \end{aligned}$$

for Polar and

13 $$\begin{aligned} \Delta ({k})_{ABM} = \Delta _{0} \begin{bmatrix} {\hat{k}_{x}+i\hat{k}_{y}} & 0 \\ 0 & {\hat{k}_{x}+i\hat{k}_{y}} \\ \end{bmatrix} = \Delta _{0} \begin{bmatrix} \sin {\theta }e^{i\phi } & 0 \\ 0 & \sin {\theta }e^{i\phi } \\ \end{bmatrix} \end{aligned}$$

for ABM phase. Here, $\Delta _0$ is the maximum gap amplitude. The vector form of order parameter i.e. the d-vector is related to the gap function as,

14 $$\begin{aligned} \hat{\Delta }({k}) = i(\vec {d}({k})\cdot \hat{\sigma })\hat{\sigma }_{y}. \end{aligned}$$

The corresponding d-vector for *purely equal-spin pairing* triplet Polar state is,

15 $$\begin{aligned} \vec {d}_{Pol}(k) = \Delta _0(0,\,-i\hat{k}_{z},\,0) \end{aligned}$$

while for the ABM state it becomes,

16 $$\begin{aligned} \vec {d}_{ABM}(k) = \Delta _0(0,\,{-i(\hat{k}_{x}+i\hat{k}_{y})},\,0). \end{aligned}$$

One notices that $\Delta _{Pol}$ is most pronounced in the z-axis but disappears in the x-y plane in the momentum space of the crystal structure of the superconductor, resulting in line nodes in the x-y plane. On the other hand, $|\Delta _{ABM}|$ has a maximum in the equatorial plane and decreases towards the poles, exhibiting point nodes exactly at north and south poles, i.e. along z-axis. The corresponding density of state acquires the form,

17 $$\begin{aligned} \rho _{Pol} = N(0)\frac{\varepsilon }{\Delta _{0}}\left( \arcsin {\left( \frac{\Delta _{0}}{\varepsilon } \right) } \Theta (|\varepsilon |-\Delta _{0})+\frac{\pi }{2}\Theta (\Delta _{0}-|\varepsilon |) \right) \end{aligned}$$

for Polar phase, and

18 $$\begin{aligned} \rho _{ABM} = N(0)\frac{\varepsilon }{\Delta _0}\ln \left| {\frac{\varepsilon +\Delta _0}{\varepsilon -\Delta _0}}\right| \end{aligned}$$

for ABM phase. Here, $N_0$ is the normal-state density of states at the Fermi level.

**Figure 2:**
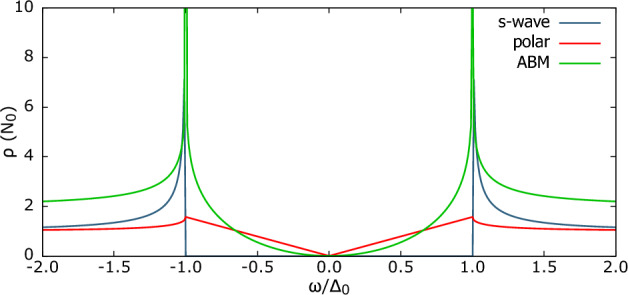
The quasiparticle density of states $\rho$ for the indicated phases: *s*-wave superconductor, *p*-wave superconductor in the Polar and ABM state, in the units of *N*(0).

The quasiparticle density of states (DoS) $\rho _i$ for these phases is shown in Fig. 2. Both the Polar and ABM state possess finite subgap ($|\omega |/\Delta _0<1$) DoS. In this range, the Polar state exhibits linear dependence, while ABM state decreases quadratically when $\omega \rightarrow 0$. Moreover, DoS for both phases possesses singularity at $|\omega /\Delta _0|=1$: in the Polar state, there is finite discontinuity cusp, whereas ABM DoS diverges logarithmically. For all the states, there is a qualitative change outside of the subgap. For reference, we also plotted the relevant density of states for *s*-wave superconductor.

### Modeling the coupling strength for anisotropic gap function

The anisotropy of the TSC gap function reflects the symmetry of the pairing state, and thus, leads to formation of Cooper pairs with directionally dependent amplitudes in the momentum space. Further, it leads to direction (angle) dependent QD-TSC tunneling amplitude $V_{k}^{TSC}$ as the QD *sees* a different gap amplitude depending on the scattering angle of an electron into the TSC lead. To incorporate this properly, one could solve a relevant set of Bogoliubov-de Gennes (BdG) equations^70–73^ for the interface of quantum dot and TSC. The full BdG treatment would provide a self-consistent spatial dependence of gap parameter $\Delta (r)$ at the QD-TSC interface. However, this approach faces some practical challenges: 1) For the chosen kind of hybrid system, interface is often ambiguous to define and experimental realization of such devices show varied interface geometry. 2) The self-consistent calculation of $\Delta (r)$ and further obtaining self-energy becomes analytically-computationally challenging and expensive. Therefore, BdG and the quasi classical approaches^74,75^ are outside of the scope of the present work.

To address this challenge, we introduce a phenomenological ansatz in which an angular ‘weight’ $W(\phi ,\theta )$ is assigned to the QD-TSC coupling matrix elements $V_{k}^{TSC}$, i.e. $V_{k} = V_{0}^{TSC} {W(\phi ,\theta )}$^60^. This is chosen such that the quasiparticle tunneling between QD and TSC has momentum in some preferred direction $k'$. For this objective, the Gaussian weight emerges as a natural choice for the reasons of: a) The quantum dot acts effectively as a point scatterer is considered to be symmetrically coupled to the finite sized lead. b) The Gaussian weight maintains the original shape of the function it is weighted by, and c) we can attribute greater or less weight closer to the mean of the Gaussian function with a single parameter.

Here we propose a weighting scheme for two configurations, depending on the orientation of the defined axis of symmetry of the TSC gap function with respect to symmetry axis of tunneling. Specifically we consider a) parallel configuration, when gap function axis aligns with the axis of tunneling and b) perpendicular configuration, for which both axes form right angle (see Fig. 1). For the parallel configuration, the self-energy is modeled as,

19 $$\begin{aligned} \boldsymbol{\Sigma }^{r}_{TSC,\parallel } = -\frac{i}{2} \Gamma _{TSC} N(\beta ) \int _{0}^{2\pi } \frac{\textrm{d}\phi }{4\pi } \int _{0}^{\pi } \textrm{d}\theta \, \sin \theta \, e^{-\beta \theta ^2} \, \textbf{FC}_i(\varepsilon , \phi , \theta ), \end{aligned}$$

and for the perpendicular case, we define

20 $$\begin{aligned} \boldsymbol{\Sigma }^{r}_{TSC, \perp } = -\frac{i}{2} \Gamma _{TSC} N(\alpha , \beta ) \int _{-\pi }^{\pi } \frac{\textrm{d}\phi }{4\pi } e^{-\alpha \phi ^2} \int _{0}^{\pi } \textrm{d}\theta \, \sin \theta \, e^{-\beta (\pi /2 - \theta )^2} \, \textbf{FC}_i(\varepsilon , \phi , \theta ). \end{aligned}$$

Here the diagonal and non-diagonal elements of the matrix $\textbf{FC}_i$, denoted as $FC_i^{D}$, $FC_i^{ND}$ respectively, acquire the following form

21 $$\begin{aligned} \begin{aligned} FC_i^{D}&= \Theta (|\varepsilon | - \Delta _0 |\gamma _i|) \frac{|\varepsilon |}{\sqrt{\varepsilon ^2 - \Delta _0^2 |\gamma _i|^2}} + \Theta (\Delta _0 |\gamma _i| - |\varepsilon |) \frac{\varepsilon }{i \sqrt{\Delta _0^2 |\gamma _i|^2 - \varepsilon ^2}}, \\ FC_i^{ND}&= \Theta (|\varepsilon | - \Delta _0 |\gamma _i|) \frac{\operatorname {sgn}(\varepsilon ) \Delta _0 \gamma _i}{\sqrt{\varepsilon ^2 - \Delta _0^2 |\gamma _i|^2}} + \Theta (\Delta _0 |\gamma _i| - |\varepsilon |) \frac{\Delta _0 \gamma _i}{i \sqrt{\Delta _0^2 |\gamma _i|^2 - \varepsilon ^2}} \end{aligned} \end{aligned}$$

such that $\gamma _{Pol} = \cos {\theta }$ for $i=Pol$, and $\gamma _{ABM} = \sin {\theta }e^{i\phi }$ for $i=ABM$. The function $\textbf{FC}_i(\varepsilon , \phi , \theta )$ is obtained by integration over normal state energy in the self-energy calculation (see Supplementary information for details). The parameters $\alpha$, $\beta$ are weights of the Gaussian distribution for azimuthal ($\phi$) and polar angle ($\theta$) respectively, such that if *b* is full width at half maximum (FWHM) of the Gaussian curve, then $\alpha , \beta \propto \frac{1}{\sqrt{b}}$. The significance of $FC_i^{ND}$ is as follows: Electrical conductance due to singlet (triplet) type of Andreev reflection is proportional to $|\boldsymbol{\rm G}^r_{12}|^2 ( |\boldsymbol{\rm G}^r_{14}|^2)$^76^, where the off-diagonal self-energy $\Sigma ^{r}_{12(14)}$ appears as an argument within the Green’s function.

The factors $N(\beta )$ and $N(\alpha , \beta )$ in Eq. (19-20) ensure proper normalization in the presence of weighting and have a form,

22 $$\begin{aligned} \frac{1}{N(\beta )} = \frac{1}{2} \int _0^{\pi } d\theta \sin \theta e^{-\beta \theta ^2} \end{aligned}$$

for parallel and

23 $$\begin{aligned} \frac{1}{N(\alpha ,\beta )} = \int _{-\pi }^{\pi } \frac{\textrm{d}\phi }{2\pi } e^{-\alpha \phi ^2} \int _0^{\pi } \frac{d\theta }{2} \sin \theta e^{-\beta (\pi /2-\theta )^2} \end{aligned}$$

for perpendicular configuration.

We note that the integration over $\phi$ is done in the interval $-\pi \le \phi \le \pi$ and not in $0 \le \phi \le 2\pi$. In average self-energy calculation (i.e. $\alpha , \beta = 0$), either choice of the integration limits gives the same result. However, since Gaussian weight in $\phi$ is symmetric in the interval $-\pi$ to $\pi$, using limits 0 to $2\pi$ produces unphysical numerical artifacts. We emphasize that in the absence of such a weighting, the self energy integral can be solved to get closed form for both the Polar and ABM case (described in the Supplementary information). Parameters $\alpha$, $\beta$ greatly influence the self-energy, which determines the Green’s function and spectral properties and therefore the tunneling coefficients and resultant charge and heat currents.

### Transport properties

The electric current flowing from one lead to the other is always mediated by the QD and can be obtained from the rate of change of occupation number in either of the lead, which is an observable related to the lesser Green’s function.^77^ Our system is assumed to be current conserving i.e. $I_{FM} =- I_{TSC}$. A positive (negative) particle (charge) current is assumed to flow from the FM lead towards the QD. The general current formula employing the non-equilibrium Green’s function formalism was obtained in Refs.^78,79^. Under the assumption of non-interacting leads, the current takes the following Landauer-like form^77^,

24 $$\begin{aligned} I=\frac{e}{h}\sum _{\sigma }\int d\varepsilon [f_{FM}(\varepsilon )-f_{TSC}(\varepsilon )]T_{\sigma }(\varepsilon ). \end{aligned}$$

Here, $T_{\sigma }(\varepsilon )$ denotes tunneling coefficient. A hybrid system with at least one superconducting lead hosts both the quasiparticle and Andreev reflection currents (involving singlet or triplet electron pair depending on the superconductor type). Therefore, the total current can be written as the sum of currents, corresponding to quasiparticle tunneling $I_{\sigma }^{\textrm{QP}}$ and Andreev current, $I_{\sigma }^{\textrm{AR}}$ as,

25 $$\begin{aligned} I= I_{\uparrow }+I_{\downarrow } = \sum _{\sigma }(I_{\sigma }^{\textrm{QP}}+I_{\sigma }^{\textrm{AR}}). \end{aligned}$$

Let $T_{FM} (T_{TSC})$ be the temperature of the *FM*(*TSC*) lead. Then their Fermi-Dirac distribution function is given by $f_{FM (TSC)}(\varepsilon ) = {[1+\exp ({(\varepsilon \pm \mu _{FM (TSC)})/k_{b}T_{FM (TSC)})]}}^{-1}$, where $-(+)$ indicates electron (hole) contribution in the lead. Then, the individual current contributions due to quasiparticle and Andreev current are;

26 $$\begin{aligned} & I_{\sigma }^{\textrm{QP}} = \frac{e}{h}\int \textrm{d}\varepsilon \left[f_{FM}(\varepsilon -\mu _{FM})-f_{TSC}(\varepsilon -\mu _{TSC})\right] T_{\sigma }^\textrm{QP}(\varepsilon ) , \end{aligned}$$

27 $$\begin{aligned} & I_{\sigma }^{\textrm{AR}}= \frac{e}{h}\int d\varepsilon \left[ f_{FM}(\varepsilon -\mu _{FM}) -f_{FM}(\varepsilon +\mu _{FM})\right] T_{\sigma }^{\textrm{AR}}(\varepsilon ). \end{aligned}$$

Here, the $T^\textrm{AR, QP}_{\sigma }$ are the tunneling coefficients, which are functions of the elements of Green’s function and acquire the form,

28 $$\begin{aligned} & T^\textrm{AR}(\varepsilon )=T^\textrm{AR}_{\uparrow }(\varepsilon )+T^\textrm{AR}_\downarrow (\varepsilon ) = \sum _{i=1,3}\sum _{j=2,4} G_{ij}^r{[\boldsymbol{\Gamma }_{FM}\boldsymbol{\rm G}^a\boldsymbol{\Gamma }_{FM}]}_{ji}, \end{aligned}$$

29 $$\begin{aligned} & T_{\uparrow (\downarrow )}^\textrm{QP}(\varepsilon )=[\boldsymbol{\rm G}^r\boldsymbol{\Gamma }_{TSC}\boldsymbol{\rm G}^a\boldsymbol{\Gamma }_{FM}]_{11(33)}. \end{aligned}$$

In the summation above, the elements *i*, *j* label the Nambu matrix elements of the Green’s function. The summation in Eq. 28 includes both the singlet ((i,j)=(1,2), (3,4)) and triplet ((i,j)=(1,4), (3,2)) Andreev reflection for the chosen Nambu basis. Similarly, the corresponding heat currents are;

30 $$\begin{aligned} & J_{\sigma }^{\textrm{QP}}= \frac{1}{h} \int \textrm{d}\varepsilon (\varepsilon -\mu _{FM} ) [f_{FM}(\varepsilon -\mu _{FM})-f_{TSC}(\varepsilon +\mu _{TSC} )]T_{\sigma }^{\textrm{QP}} (\varepsilon ), \end{aligned}$$

31 $$\begin{aligned} & J_{\sigma }^{\textrm{AR}}=\frac{-\mu _{FM}}{h} \int \textrm{d}\varepsilon [f_{FM}(\varepsilon -\mu _{FM} )-f_{FM}(\varepsilon +\mu _{FM} )] T_{\sigma }^{\textrm{AR}} (\varepsilon ). \end{aligned}$$

#### Linear response regime

Let us express the chemical potential of the FM lead with respect to the superconductor’s chemical potential, i.e. $\mu _{FM}=\mu +\delta \mu$. We assume the superconducting lead is grounded i.e. $\mu _{TSC}\equiv \mu =0$. Similarly the temperature of the FM lead is measured with respect to the TSC reservoir as $T_{FM}=T+\delta T$, with $T_{TSC}\equiv T$. Within linear response approximation, i.e. for $\delta \mu /k_BT\ll 1$ and $\delta T/T\ll 1$, we expand particle/charge and heat currents with respect to the driving forces $\delta \mu$ and $\delta T/T$ up to linear order to obtain:

32 $$\begin{aligned} \begin{pmatrix} I\\ J \end{pmatrix} = \sum _{\sigma } \begin{pmatrix} e^2L_{0\sigma } & & eL_{1\sigma }\\ eL_{1\sigma }& & L_{2\sigma } \end{pmatrix} \begin{pmatrix} \delta \mu \\ \delta T/T \end{pmatrix} = \sum _{\sigma }\mathbb {L}_\sigma \begin{pmatrix} \delta \mu \\ \delta T/T \end{pmatrix} \end{aligned}$$

with

33 $$\begin{aligned} L_{n\sigma }=\frac{1}{h}\int d\varepsilon (\varepsilon -\mu )^{n}\left( -\frac{\partial f}{\partial \varepsilon }\right) T_{\sigma }^{X}(\varepsilon ) \end{aligned}$$

for $X = QP, AR$. In the linear response regime, it can be verified that the Onsager matrix takes the form:

34 $$\begin{aligned} \mathbb {L}_{\sigma }= \frac{1}{h}\int d\varepsilon \left( -\frac{\partial f}{\partial \varepsilon }\right) \begin{pmatrix} (2T_{\sigma }^{AR}+T_{\sigma }^{QP}) & & (\varepsilon -\mu )T_{\sigma }^{QP}\\ (\varepsilon -\mu )T_{\sigma }^{QP}& & (\varepsilon -\mu )^{2}T_{\sigma }^{QP} \end{pmatrix} \end{aligned}.$$

#### Thermoelectric coefficients

The thermoelectric transport coefficients of electrical conductance *G*, thermopower (Seebeck coefficient) *S*, and electronic contribution to thermal conductance $\kappa$ can be expressed using Eq. 34. The electrical conductance is defined as the charge current per unit voltage bias between the isothermal reservoirs,

35 $$\begin{aligned} G = \left( \frac{eI}{\delta \mu }\right) _{\begin{array}{c} \delta T=0 \end{array}}= \sum _{\sigma }e^2L_{0\sigma }. \end{aligned}$$

The thermopower *S* is defined as the ratio of the voltage drop $\delta \mu$ generated between two reservoirs by the temperature difference $\delta T$, measured in the absence of charge current,

36 $$\begin{aligned} S= -\left( \frac{\delta \mu _{}}{e\delta T }\right) _{\begin{array}{c} I=0 \end{array}} = -\frac{1}{eT}\frac{\sum _{\sigma }L_{1\sigma }}{\sum _{\sigma }L_{0\sigma }}. \end{aligned}$$

In turn, the thermal conductance is defined as,

37 $$\begin{aligned} \kappa =\left( \frac{J}{\delta T}\right) _{\begin{array}{c} I=0 \end{array}}= \frac{1}{T}\left( \sum _{\sigma }L_{2\sigma }-\frac{[\sum _{\sigma }L_{1\sigma }]^{2}}{\sum _{\sigma }L_{0\sigma }}\right) \end{aligned}$$

The corresponding figure of merit, *ZT*, measures the efficiency of heat current to be converted into electrical current and is defined as $ZT=S^2G/\kappa$.

## Numerical results and discussion

In this section, we present the thermoelectric transport coefficients- electrical conductance, thermopower, thermal conductance, and figure of merit as function of the quantum dot’s energy level. The results are divided into two distinct parts: A) Polar state with average (unweighted) self-energy (for-benchmarking): we examine the influence of the system parameters, specifically the TSC-QD coupling relative to the FM-QD coupling and the FM spin-polarization (*p*), on thermoelectric properties when only the bare average self-energy i.e. without weighting, is considered. These parameters are independent of the spatial differential coupling of TSC. Variation in polarization emphasizes the role of ferromagnet in the device. The relative coupling shows how transport behaves if anisotropic gap were treated like a *s*-wave gap, hence providing a benchmark for comparison. Note that the relative coupling is also dependent on substrate properties such as its conductance, thickness, device dimensions, lead-substrate hybridization which are independent of the superconducting phase and hence important for benchmarking. B) Effect of anisotropic gap and angular weighting: Next and central to this article is varying the Gaussian weighting parameters (s) and the rotating axis of TSC gap symmetry with respect to QD’s axis of tunneling. We explicitly compute the self-energy for these parameters.

While understanding the transport properties, we make the following assumptions: 1) The quantum dot is treated as a point scatterer. 2) Only the angle at which an electron approaches from (to) QD to (from) TSC lead plays role in capturing the effect of anisotropy of the TSC gap. However, the particle tunneling angle between the FM lead and QD is irrelevant. 3) There is no loss of generality in assuming both leads to be 3 dimensional. 4) All energies are measured in units of $\Delta _0$, unless otherwise mentioned. The results are presented within linear response regime. As auxiliary figures, the tunneling coefficient for quasiparticle, Andreev reflection and the self-energies are presented in supplementary information.

### Transport coefficients for Polar state under ‘average’ QD-TSC coupling

When we refer to *average* self-energy, we assume that the TSC gap symmetry axis does not have a preferred orientation with respect to the quantum dot tunneling axis, so that all orientations are treated as equally probable. Such a situation can be attributed to polycrystalline sample, in which the grains’ superconducting gap symmetry axes are randomly oriented. As a result, the effective gap function is seen by the tunneling electrons as the average over the grains. This assumption holds exactly for *s*-wave superconductor. Under this assumption (which clearly has phenomenological limitations), electron tunneling between the QD-TSC experiences the same average coupling regardless of its momentum direction. Equivalently, the hybridization of QD energy level is unaffected by the spatial variation of the gap function and self-energy takes on its ‘average’ value. In our formalism, it simply means that no weighting is applied, i.e. $\alpha , \beta =0$.

**Figure 3:**
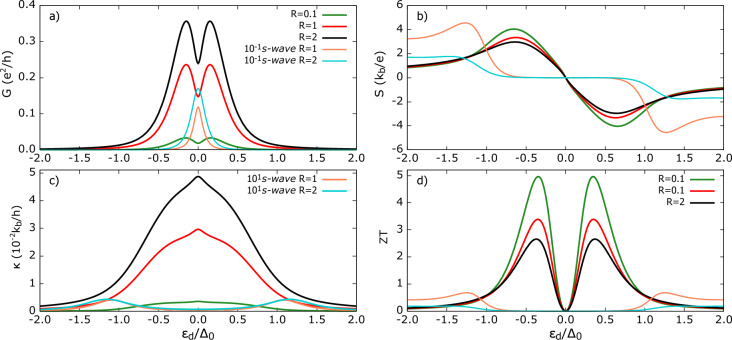
Thermoelectric coefficients for TSC in Polar state and in the absence of spatial weighting to self-energy. (**a**) electrical conductance *G* (**b**) thermopower *S* (**c**) thermal conductance $\kappa$ (**d**) figure of merit *ZT* as a function of dot’s energy level for varied QD-TSC coupling parameter *R*. Other parameters are: $U=0$, $\Gamma _{FM}=0.1\Delta _0$, $R=\Gamma _{TSC} / \Gamma _{FM}$, $k_bT=0.1\Delta _0$, $p=0.5$. For comparison, transport coefficients shown for hybrid with *s*-wave superconductor with the same parameters.

Fig. 3 shows the transport coefficients for QD-TSC in Polar state. We define $R= \Gamma _{TSC}/\Gamma _{FM}$ for a fixed value of $\Gamma _{FM}$. Here $\Gamma _{FM}$, $\Gamma _{TSC}$ are coupling strengths of the QD to FM and TSC leads. When $R=1$, both leads are equally coupled. Decreasing *R* means weakening the hybridization of QD energy level due to TSC lead compared to FM.

In Fig. 3 a), the conductance *G* has resonances at $\varepsilon _{d} \approx \pm 0.15\Delta _0$ and a local minimum at $\varepsilon _{d} = 0$ being a characteristic feature of the Polar state. The growth of *G* with increasing R is monotonic but nonlinear. As seen in Fig. 2, the density of states, $\rho _{Pol}$ has a node ($\rho _{Pol}$=0) at $\omega /\Delta _0 = 0$, resulting in the observed local minimum. (Recall that we set the chemical potential of the TSC to $\mu _{TSC}=0$, therefore conductance minimum matches the DoS minimum). In the absence of weighting, we have $\Sigma _{14}^{r}=0$, and therefore the Andreev reflection processes are totally suppressed, and thus the conductance is of purely quasiparticle origin.

The QP tunneling coefficient $T^{QP}$ (see Fig. 1 in supplementary information), for $\varepsilon _d=0$, displays increasing peak values, while the overall shape remains unchanged. The rise in area under $T^{QP}(\varepsilon )$ with *R*, manifests in the higher conductance near particle-hole symmetry point. However, the amplitude begins to saturate as we move towards the subgap edges ($|\varepsilon _d|=0.5-1$) and for $R>1$. Although quasiparticles over wider range of energy gain a finite probability to tunnel, the net charge flow does not increase proportionally. However, the area under $T^{QP}$ increases with *R* regardless of the dot’s energy level, leading to overall rise of charge conductance. This behavior is consistent with the level broadening of the QD energy level, which allows electrons with a wider range of energy to tunnel to the superconductor.

For comparison, we also present these thermoelectric quantities for superconductor in conventional *s*-wave state. In this case, conductance exhibits a single prominent resonance peak centered at $\varepsilon _d=0$, ascribed to the Andreev reflection^80–83^. Our analysis of individual contributions shows that, for the *s*-wave case there is $\approx 10^4$ difference between $G^{AR}$ and $G^{QP}$ (with $G^{AR} > G^{QP}$). Thus, in the present hybrid, Polar state’s QP conductance magnitude is comparable to the *s*-wave AR conductance, despite the low temperature ($k_{b}T=0.1\Delta _0$).

Fig. 3 b) presents the thermopower *S*. A key observation is a steep increase of *S* within the subgap region, and *S* attains maximum approximately at the same QD energy level ($\varepsilon _d\approx 0.6\Delta_0$), only slightly shifting outward with *R*. A finite, linearly growing quasiparticle DoS in the subgap manifests in finite and smoothly rising thermopower. Moreover, *S* changes sign at $\varepsilon _{d}=0$, where positive (negative) Seebeck coefficient indicates that the holes (electrons) dominate thermally induced voltage buildup. The largest |*S*| is obtained for the smallest value of *R*, which can be understood as follows: for the weakly coupled TSC , electrical conductance is the least and by definition, $S \propto 1/L_{11}$ with $L_{11} = G$. Therefore, by Eq. 36, the Onsagar matrix element $L_{12}$, which reflects strength of driving electrical current induced by temperature difference $\delta T=T_{FM}-T_{TSC}$, increases only weekly with *R* . More physically, it can be understood by referring that thermopower is defined under the condition of vanishing charge current. Therefore, as *G* increases with increasing *R*, a smaller bias voltage has to be applied to compensate the current induced by the temperature difference, consequently lowering |*S*| with increasing *R* . Outside the subgap region, *S* decreases smoothly owing to the qualitative change in DoS which does not differ sharply in magnitude (Fig. 2). This behavior is in striking contrast to the *s*-wave state for which the thermopower is substantially higher compared to TSC in the Polar state. The conductance $G=L_{11}$ for the Polar state is still significantly greater than the *s*-wave state for $|\varepsilon _d|>>\Delta _0$. Therefore, a smaller compensating voltage is required to be applied to cancel the thermally generated current.

Fig. 3c) shows the thermal conductance $\kappa$. For all values of *R*, $\kappa$ exhibits a peculiar non-monotonic behavior. Over most of the $\varepsilon _d$ range, the curve is convex. However, as $\varepsilon _d\rightarrow (\pm ) 0$, it becomes concave. This reversal of curvature is pronounced, i.e. the radius of concave curvature is small for a weakly coupled TSC and this effect weakens (i.e. the radius of curvature is large) as *R* increases. This concave region indicates the slower rate of increase of $\kappa (|\varepsilon _{d}|)$ in the range $|\varepsilon _{d}| \lessapprox 0.2\Delta_0$, due to the sudden drop in the quasiparticle DoS at the particle-hole symmetry point, which limits the available channels for heat transport. Interestingly, despite electrical conductance reaching local minimum at $\varepsilon _{d}=0$, the $\kappa$ shows sharp maximum, which is attributed to the bipolar effect^84^. Elaborating on the bipolar effect: at the particle-hole symmetry point ($\varepsilon _d = 0$ for non-interacting QD), the energy required to add an electron into or remove an electron from QD is same relative to the Fermi energy of the lead (assumed to be the zero level). Therefore, in linear response, the electric current due to electrons is exactly canceled by the current carried by holes. The heat current, however, behaves differently as both electrons and holes transfer energy in the same direction and take heat energy away from the hot lead towards the cold lead. Consequently, their heat contributions add constructively, and this effect produces the central peak in the thermal conductance. From Eqs. (37) and (33), $\kappa \propto {(\varepsilon -\mu )}^{2} T^{QP}$, and at $\varepsilon _d=0$, the tunneling coefficient $T^{QP}$ is symmetric about $\varepsilon =0$. Therefore, with $\mu =0$, the electrons (above the chemical potential, $\varepsilon >0$) and holes ($\varepsilon <0$) contribute additively, giving rise to bipolar enhancement.

Compared with the *s*-wave state, $\kappa$ for the Polar TSC is enhanced by $\approx 10^2$. The $\rho _{Pol}$ outside of the gap is slightly lower than the $\rho _{s-wave}$ state (see Fig.2); hence, prima facie, one might expect comparable $\kappa$. However, this staggering difference can be understood as follows: in the *s*-wave case, Andreev conductance dominates the total charge conductance and primarily occurs in the subgap, but it does not directly contribute to heat conductance in the linear response regime. Therefore, $\kappa _{s-wave}$ is suppressed within the gap. In turn, for the Polar TSC, a finite quasiparticle DoS enables heat transport, which results in pronounced $\kappa$. Moreover, $\kappa$ for the Polar TSC does not depend on the AR contribution to charge conductance, as it is absent here (opposite to the *s*-wave scenario). Interestingly, even outside the subgap, $\kappa$ for the *s*-wave and for $R=1$ remains less than $\kappa$ for the Polar TSC at $R=0.1$. For the *s*-wave hybrid, the broadening of the dot’s energy level only weakly depends on *R*. In contrast, for the Polar state, the energy level broadening varies strongly with coupling. As a result, a wider range of TSC states contribute to $\kappa$ for a given $\varepsilon _d$. This explains the stronger increase of $\kappa$ for $|\varepsilon _d|>\Delta _0$. Finally, the *ZT* shown in Fig. 3d) exhibits two resonance peaks and vanishes at $\varepsilon _d=0$ due to the vanishing thermopower. *ZT* is greatest for stronger QD-TSC coupling, and therefore a higher *R* is desirable for the efficient conversion of the thermal gradient into the generated power in the device. The increase of *ZT* with *R* is mainly attributed to the decrease of heat conductance. The $ZT(s-wave)$ is significantly smaller ($ZT(Pol)\approx 4 ZT (s-wave)$) for the same set of parameters and remains finite outside the superconducting gap. Therefore, Polar-state-based materials are likely to exhibit superior thermoelectric performance.

**Figure 4:**
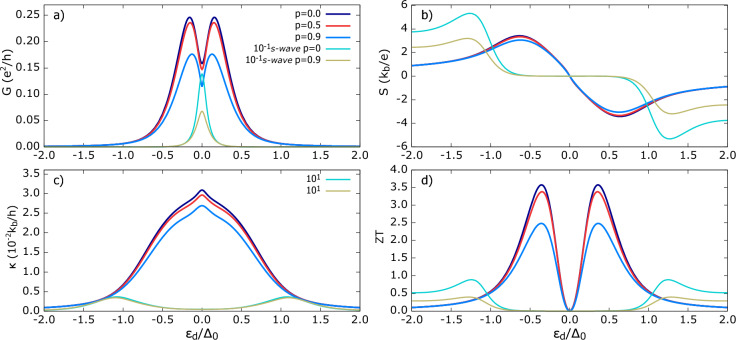
Thermoelectric coefficients for TSC in Polar state and in the absence of spatial weighting to self-energy (**a**) electrical conductance *G* (**b**) thermopower *S* (**c**) thermal conductance $\kappa$ (**d**) figure of merit *ZT* as a function of dot’s energy level for different polarization of FM lead. Other parameters are: $U=0$, $\Gamma _{FM},\Gamma _{TSC} =0.1\Delta _0$, $k_bT=0.1\Delta _0$, $p$ as indicated in figures. For comparison, transport coefficients shown for hybrid with *s*-wave superconductor with the same parameters.

Fig. 4 presents the influence of spin polarization *p* of the ferromagnetic lead. As *p* increases, the fraction of minority-spin ($\downarrow$) carriers decreases. In Fig. 4a), the electrical conductance drops significantly with increasing *p*. This is consistent with Polar state’s electrical conductance in the ‘average’ self-energy case, where $G=G_{QP}$ and no spin flip processes are allowed in our model. The majority carrier conductance remains unchanged by variations in *p*, while the minority carrier tunneling is suppressed. This arises because, in the superconductor, both spin $\uparrow$ and $\downarrow$ states are equally present and couple equally to the QD. For the given set of parameters, the Polar state based hybrid is noticeably less sensitive to polarization changes than the *s*-wave state. This is evident from their relative change in $G_{max}$ for $p=0,0.9$. This can be attributed to the fact that conventional Andreev tunneling requires spin pairing of electrons with both spin orientations, in contrast to the quasiparticle current, which involves spin-conserving direct transfer of individual electrons. Thus, Andreev reflection contribution to the conductance decreases steadily for *s*-wave case with increasing *p* and vanishes as *p* achieves its maximal value ($p=1$). In turn, for the Polar TSC, there is still an active spin-up channel for $p=1$, the conduction of which doesn’t depend on *p*. Thus, for $p=1$, the conductance for Polar TSC case is approximately halved with respect to the corresponding value for $p=0$. Typically, $\varepsilon _d$ is tunable by an external gate bias^85,86^. The broader resonance $G_{Pol}(\varepsilon _d)$ implies that finite conductance persists over a wider range and is less sensitive to parameter variation in $\varepsilon _d$ than the *s*-wave case. This feature might be advantageous for device operation under parameter fluctuations.

The thermopower *S*, shown in Fig. 4b), remains largely unaffected by increasing *p*, except for a slight change near the maxima. This robustness occurs despite a significant decrease in electrical conductance and can be understood as follows: with increasing *p*, both $L_{11} (=G)$ and $L_{12}$ decrease roughly at the same rate with *p*, which leads to a weak *p*-dependence of *S*. In the absence of Andreev conductance, the thermopower depends on the asymmetry of $T^{QP}(\varepsilon )$ around $\varepsilon =0$ (see supplementary information Fig. S1). Varying polarization does change the absolute value of $T^{QP}(\varepsilon )$; its intensity decreases with increasing *p* due to fewer available minority spin states. However, it does not significantly alter this asymmetry, explaining the indifference to varying *p*. On the other hand, there is a significant difference for the conventional *s*-wave state, with *S* following a reverse trend compared to *S* for the Polar TSC, a trend influenced by the covert influence of Andreev reflection conductance^80,84^. The thermal conductance $\kappa$, shown in Fig. 4c), like the charge conductance, is renormalized by the *p* variation without any change in its functional shape. This is a qualitatively distinct effect from that which is induced by the coupling strength *R*. The significance of this lies in the fact that, despite $L_{12,22}$ components having an implicit dependence on *p*, through the self-energy $\Sigma _{11}^r$, the $\kappa$ profile remains indifferent. This observation is experimentally relevant since, in the absence of a magnetic field, the intrinsic polarization of the FM lead does not change the shape, allowing theoretical predictions for different polarization values. However, greater *p* is detrimental to the efficiency of the system, as reflected in *ZT* in Fig. 4d), which ranges between 3-4. Though the components of *ZT*, i.e. *G*, *S*, and $\kappa$, maintain their spectral shape with increasing *p*, highly polarized leads may limit device performance.

## Effect of gap anisotropy on transport properties

We now discuss the behavior of thermoelectric transport properties of the device based on a) superconducting phase (Polar or ABM) b) relative configuration of the superconducting lead (parallel or perpendicular) and c) weighting factor, as described in the Sec. Modeling the coupling strength for anisotropic gap function.

### Polar state-parallel configuration

**Figure 5:**
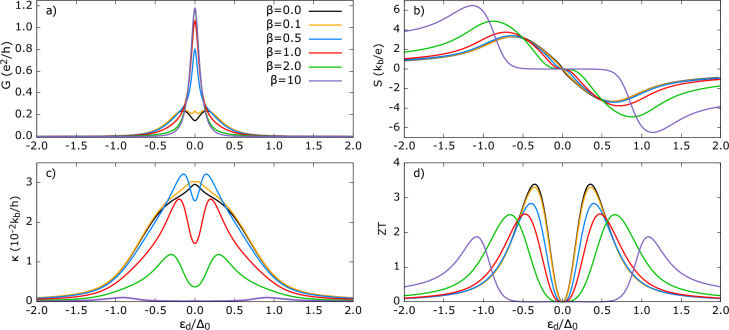
Thermoelectric coefficients for Polar state in *parallel* configuration. (**a**) conductance *G*, (**b**) Thermopower *S* (**c**) Thermal conductance $$\kappa$$ (**d**) *ZT*. The other parameters: U=0, $$\Gamma _{FM}=$$$$\Gamma _{TSC}=0.1\Delta_0$$, $$k_bT=0.1\Delta_0$$, $$p=0.5$$.

For $\beta =0$, i.e. in the absence of weighting, the QD couples equally to all momentum directions $\vec {k}$ of the tunneling electron. As seen in the Fig. 5a), a valley appears at particle-hole symmetry point $\varepsilon _d=0$, and overall, we have $G=G_{QP}$. This case is equivalent to the average conductance that we discussed in the Sec. Transport coefficients for Polar state under ‘average’ QD-TSC coupling. For even a small finite $\beta (\approx 0.1)$, a midgap peak starts to emerge at $\varepsilon _d=0$. There are two distinct peaks in *G* close to $|\varepsilon _{d}| \approx 0.15$ with amplitudes comparable to the midgap peak. These lateral peaks primarily originate from quasiparticle tunneling. In the parallel configuration, an increase in $\beta$ phenomenologically indicates that the QD-TSC coupling becomes stronger when $\theta$ is close to 0. For the Polar state in parallel configuration, this is also the region where $\Delta (k)$ attains its larger value and has a maximum at $\theta =0,\pi$. This can be interpreted as an increasing probability of a triplet pair tunneling to (from) the superconducting condensate, in a direction confined to a smaller solid angle around $\theta =0$. This effect is independent of the absolute value of the momentum. Consequently, these same tunneling modes are the ones that most effectively participate in triplet Andreev reflection as $\beta$ increases. In Fig. 5, the midgap peak emerges stronger with increasing $\beta$, and we emphasize that it originates from the *triplet Andreev* reflection. Apart from the intrinsic triplet pairing of the TSC lead considered here, equal-spin (triplet) AR can also arise in systems with conventional *s*-wave superconductors through spin-mixing mechanisms. For example, intra-dot spin-flip processes due to exchange interaction can generate equal-spin states^87,88^. In low-dimensional or interface-engineered systems through Rashba spin-orbit coupling, possibly combined with spin-flip scattering or broken time-reversal symmetry can induce triplet correlations^89–91^. Similarly, singlet-triplet conversion may also occur via proximity effects in hybrid structures with noncollinear exchange fields^92^.

Notice that for $\beta >0$ the self-energy, $[\Sigma _{TSC,\parallel }^{r}]_{14}$ (see Supplementary information Fig. S6), becomes nonzero, directly indicating an onset of triplet Andreev reflection processes, resulting in a finite $T^{AR}$ (see Supplementary information Fig. S2), and therefore contributing to *G*. This feature is absent in Fig. 3 and emphasizes that neglecting the anisotropic QD-TSC coupling in the device modeling can lead one to miss crucial qualitative effects. The three-peak structure of charge conductance gradually disappears with increasing $\beta$, merging into a single peak. The amplitude of the peak increases with $\beta$ until it saturates for a certain value of $\beta$ (around 2). Interestingly, the peak width decreases with increasing $\beta$, which can be attributed to the diminishing role of QP tunneling.

Indeed, analyzing the individual contributions to the tunneling processes corresponding to the QP and triplet AR processes (see Supplementary information Fig. S2), it becomes clear that the rise of triplet AR contribution to *G* comes at the expense of the decrease in the QP contribution. For $0.2< |\varepsilon _d| < 1$, the conductance is larger for smaller $\beta$, while near particle-hole symmetry ($|\varepsilon _d| \lesssim 0.2$), the trend reverses and *G* becomes larger for higher $\beta$. This non-monotonic variation of $G(\varepsilon _d)$ with $\beta$ reflects the dependence of tunneling amplitudes $T^{AR}$ and $T^{QP}$ on $\beta$ through the corresponding self-energies ($\Sigma ^r_{11}$ and $\Sigma ^r_{14}$). The real and imaginary components of the self-energy evolve in a complex manner with $\beta$ (see Supplementary information Fig. S6), making detailed tracing of the peak behavior non-trivial.

At low temperature (here, $k_bT = 0.1\Delta _0$), the Andreev reflection in *s*-wave superconducting hybrid can supersede the quasiparticle conductance by $10^2-10^4$ orders of magnitude (see Supplementary information in ref.^84^). For the Polar state, the triplet Andreev reflection is dominant by $10^1-10^2$ but behaves differently. Thus increasing $\beta$ enhances the dissipationless triplet Andreev conductance. However, this enhancement is achieved at the cost of losing distinctive qualitative features of Polar state, such as pronounced subgap conductance between $0.5<|\varepsilon _d|<1.0$, apart from the Andreev peak.

 Specifically, for larger values of $\beta$, the form of *G* starts matching the hybrid with the *s*-wave superconductor^80,81^, which diminishes its significance as a distinguishing signature. This underscores the tradeoff between maximizing Andreev conductance and preserving distinguishing features of the Polar state in observables.

The thermopower |*S*| response, as shown in Fig. 5b), demonstrates how tuning $\beta$ influences both magnitude and profile of *S* across $\varepsilon _d$. First let’s discuss the behavior within the range $|\varepsilon _{d}| < 0.5$, where *S* is smaller for greater $\beta$ values. In the range $\approx -0.2< \varepsilon _{d} < 0.2$, the denominator, $L_{11} (= G_{QP}+G_{AR})$, increases with $\beta$ but decreases beyond this range. However, $|L_{12}|$, a measure of diffusive particle transport, decreases almost monotonically throughout $\varepsilon _d$. The profound influence of triplet AR processes on the thermopower is revealed, especially for larger values of parameter $\beta$ and $|\varepsilon _d|<0.5$. In this case we have $\frac{dS}{d\varepsilon _d} (\beta ) \approx 0$. This feature results from the enhanced Andreev process and simultaneous suppression of QP tunneling. Thus, the denominator of *S* is mainly governed by $L_{11}\approx G^{AR}$. As the triplet AR processes do not contribute to the current induced by temperature difference, i.e. $L_{12}$ is determined solely by the QP tunneling coefficient $T^{QP}$, the thermopower becomes strongly suppressed in the considered dot’s energy level range. This flattening underscores the intrinsic particle-hole symmetry due to Andreev reflection induced by the weighted QD-TSC coupling, which does not manifest in its absence.

Now, at higher QD energies $|\varepsilon _d|\gtrsim 0.5$, where the Andreev conductance is subdued, the thermopower increases with increasing $\beta$, leading to a larger voltage developed across the leads. *S* curves exhibit a crossover-a characteristic of the parallel configuration. In this regime, moving towards higher $\varepsilon _d$, the approximation $S(\varepsilon _d) \approx \frac{\int \frac{df}{d\varepsilon } \varepsilon T^{QP}\!(\beta ,\varepsilon )}{\int \frac{df}{d\varepsilon } T^{QP}\!(\beta ,\varepsilon )}$ becomes increasingly valid, representing the mean value of energy $\varepsilon$ of quasiparticles participating in transport, weighted by $T^{QP}$.

The shift of the maximum of |*S*| towards higher $\varepsilon _d$ can be explained by considering the interplay of $L_{12}$ and $L_{11}=G$ components with varying $\beta$. Both components decrease with increasing $\beta$ and with increasing $\varepsilon _d$. The dependence on $\varepsilon _d$ reflects the overlap of $T^{QP}$ with the Fermi function-the further the level is from the Fermi energy, the smaller the number of (high-energetic) quasiparticles available for transport (which could potentially contribute significantly to $L_{12}$). As $\beta$ increases, the conductance decreases faster when moving $\varepsilon _d$ away from $\varepsilon _d=0$ (see Fig. 5a). For $\varepsilon _d\approx 0.5\Delta _0$ the rate of decrease of $L_{12}$ is almost the same as for $L_{11}$, resulting in essentially no change in *S* with varying $\beta$. However, for larger $\varepsilon _d$ this changes: $L_{12}$ decreases more slowly with $\beta$ than $L_{11}$, as $L_{12}$ is weighted by the quasiparticle energy. As a result, *S* increases with $\beta$ for $|\varepsilon _d|>0.5\Delta _0$.

Similar to the thermopower, the thermal conductance $\kappa$ (Fig. 5c) is directly dominated by the quasiparticle flow. A finite $\beta$ suppresses the central maxima in $\kappa$, which can be understood as the reduced access of heat-carrying quasiparticles to the subgap ($-1<\varepsilon _{d}<1$) density of states of the bulk superconductor. At $\varepsilon _d=0$, the valley in tunneling coefficient $T^{QP}$ (see Supplementary information Fig. S2) near $\varepsilon =0$ broadens with increasing $\beta$. This weakens the *bipolar effect*–that is, the additive effect of both electrons and holes in thermal conduction–therefore reducing $\kappa$ in the vicinity of $\varepsilon _d=0$.

A key distinction of $\kappa$ compared to conventional *s*-wave^80,81,84^ superconductors is that a) the maximum of $\kappa$ for zero Coulomb correlation ($U=0$), $\kappa _{max}$, appears within the subgap region, provided the subgap is defined by the maximum of TSC gap ($\varepsilon _d<\Delta _0$); and b) at a chosen low temperature ($k_bT=0.1\Delta _0$), $\kappa$ is enhanced by up to $10^{2}$ orders of magnitude.

The compiled effect of *G*, *S*, and $\kappa$ on the system’s efficiency, measured in *ZT* (Fig. 5d), reveals a non-trivial trend (particularly compared to Fig. 3) as a result of qualitative changes in these transport coefficients. Here, the *ZT* peak ranges between 2–4 as $\beta$ varies from 0–10. Thermopower largely determines the position of *ZT* resonance on the $\varepsilon _d$ axis. Since the maximum *ZT* occurs for $\beta =0$, minimizing Andreev reflection enhances thermoelectric efficiency. Also, the relation between AR and position of *ZT* can be understood as follows: in the linear response regime, $ZT \propto {L_{12}^2}/({L_{22}L_{11}-L_{12}^2})$, and AR contributes directly only to $L_{11}$. Therefore, for fixed $\beta$, larger AR enhances the denominator and suppresses *ZT*. Crucially, $G^{AR}=G^{AR}(\varepsilon _d)$; the spectral distribution of AR modifies the denominator of *ZT*, and its maximum shifts to where AR is relatively weaker compared to the quasiparticle transport (see Supplementary information Fig. S8.)

### Polar state-perpendicular configuration

**Figure 6:**
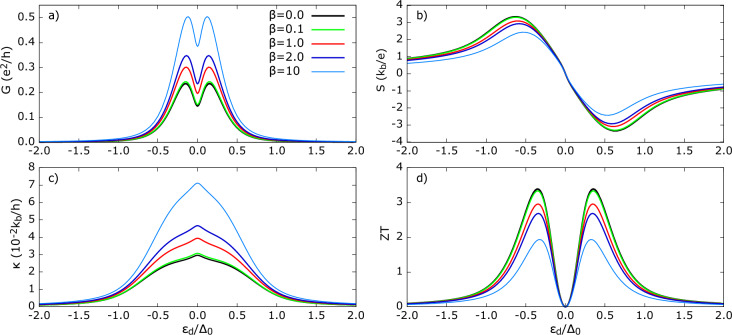
Thermoelectric coefficients for Polar state in *perpendicular* configuration (**a**) conductance *G*, (**b**) thermopower *S* (**c**) thermal conductance $\kappa$ (**d**) figure of merit *ZT* . The other parameters: U=0, $\Gamma _{FM}=\Gamma _{TSC}=0.1\Delta_0$, $k_bT=0.1\Delta_0$, $p=0.5$. Gaussian weight parameter for azimuthal angle $\alpha$ is not mentioned in the figure because it does not affect the transport properties. Explanation given in the text.

Now we analyze the case where the polar axis of symmetry of the TSC gap is oriented perpendicularly, thereby placing the quantum dot in the equatorial plane of the superconductor’s momentum space. In this configuration (Fig. 6), one would plausibly expect the Gaussian weight over the azimuthal angle $\alpha$ to influence transport. This expectation arises because, for any cross-section of either lobe of the *p*-wave gap structure, quasiparticles with momentum in a certain confined range of azimuthal angles are likely to be more strongly coupled to the QD than others. But, due to the lack of explicit $\phi$ dependence of $\Delta _{Pol}$, the normalization factor (Eq. 23) and the integral over $\phi$ in the self-energy (Eq. 20) exactly cancel out the effect of $\alpha$. However, we emphasize that this is a special case when the quantum dot is exactly in the equatorial plane of the *momentum space* of TSC.

The transport coefficients in the perpendicular configuration are qualitatively different from the parallel case. As shown in Fig. 6a), the conductance *G* increases with $\beta$, and no resonance peak appears at $\varepsilon _d=0$. This is because, in the perpendicular configuration, the off-diagonal self-energy, $\Sigma _{14}^r=0$ (see Fig. 6 in Supplementary information), vanishes, thus suppressing the Andreev reflection.

Notably, the absence of AR differs from the case of ‘average’ QD-TSC coupling discussed in the Sect. Transport coefficients for Polar state under ‘average’ QD-TSC coupling. Here, the Gaussian weight is now symmetric about $\theta =\pi /2$ [see Eq. 20]. This symmetry implies that for every Cooper pair that could form by AR from electrons with momentum directions in angles $\theta _i$ and $\pi +\theta _i$, there exists an equally weighted process involving electrons in angles $\pi -\theta _i$ and $-\theta _i$.

In the integrand of $\Sigma _{14,\perp }^r$ over $\theta$, the numerator at some $\theta _i \in (0, \pi )$ is $\cos {\theta _i}\sin {\theta _i} \exp {(-\beta (\pi /2-\theta _i)^2)}$ and, similarly, the corresponding contribution at $(\pi -\theta _i)$ is, $\cos {(\pi -\theta _i)}\sin {(\pi -\theta _i)}\exp {(-\beta (\pi /2-(\pi -\theta _i))^2)}= -\cos {\theta _i}\sin {\theta _i}\exp {(-\beta (-\pi /2+\theta _i)^2)}$. This relation, which is manifestly independent of $\varepsilon$, exactly cancels in the integration, hence leading to $\Sigma _{14}^{r}=0.$

Phenomenologically, this does not mean that the Andreev reflection cannot take place locally. But rather, at equilibrium, there being a destructive interference of Andreev reflection between Cooper pairs that could tunnel from QD to TSC and vice versa, results in the suppression of the net Andreev reflection conductance.

Notice also that an electron with a positive $k_z$ component, scattered to the positive $+z$ lobe (*north* lobe), experiences a positive gap function $\Delta (k)>0$, whereas an electron with negative $k_z$ (*south* lobe) feels a negative gap function $\Delta (k)<0$. Analogously, the corresponding reflected holes experience negative/positive gap functions, respectively. In the perpendicular configuration, scattering of quasiparticles to the *north* and *south* lobes is equally probable, and taking into account the $\pi$ shift between the $+z$ and $-z$ gap functions, complete destructive quantum interference emerges (as mentioned above), leading to cancellation of the net Andreev current. Therefore, $G=G_{QP}|_{Pol,\perp }$ and the quasiparticle transport grows with $\beta$. Raising $\beta$ means narrowing the angular distribution of electrons tunneling at the QD-TSC interface into a smaller solid angle centered at $\theta =\frac{\pi }{2}$. Since TSC gap node ($\Delta (k) =0$) exists at $\theta =\frac{\pi }{2}$, quasiparticle transport dominates the conduction. While the Andreev reflection can take place for a fixed $\theta$, its net contribution to the conductance vanishes in angular integration due to symmetry. Away from $\theta =\frac{\pi }{2}$, for any fixed $\theta _c$, the tunneling electron encounters a finite and stronger $\Delta (k)$, where, locally in $\theta$, Andreev reflection would be the preferred tunneling mechanism.

Furthermore, $\beta$ variation brings only renormalization effect on the conductance magnitude and preserves the shape within the subgap region $\approx |\varepsilon | \lesssim 0.5\Delta_0$. This implies a scaling function of the form $G_{\beta _2} \approx c(\beta _1,\beta _2) G_{\beta _1}$ exists; particularly, each curve can be related to that for $\beta =0$ by a multiplying factor. The Onsager element $L_{11}$ is solely determined by QP tunneling. In Fig. 6b), the thermopower exhibits a monotonous, meager variation with increasing $\beta$. Variation in *S* stems from the term $\varepsilon -\mu$ in $L_{12}$, which ascribes more weight (here $\varepsilon$) to larger energy in the Fermi sea of the leads given by the expression $S(\varepsilon _d) \propto \frac{\int d\varepsilon \frac{df}{d\varepsilon }\varepsilon T^{QP}\!(\beta ,\varepsilon )}{\int d\varepsilon \frac{df}{d\varepsilon } T^{QP}\!(\beta ,\varepsilon )}|$ (see Fig. S3 in Supplementary information). Therefore, the insignificant *S* variation with $\beta$ directly originates from diminishing variation in the rate at which $T^{QP}$ varies as a function of $\varepsilon$ as $\beta$ is varied.

Deep inside the subgap, *S* shows a linear growth; thus, we infer that weighting does not destroy this feature observed in the Fig. 3c). Such linear growth is not observed in hybrids with an *s*-wave state. Experimentally, thermopower measurements can potentially be used to determine the axis of symmetry of the TSC gap function in the device and to obtain the condition of maximum AR. By rotating the crystal axis of symmetry of TSC lead with respect to the QD, one can monitor the *S* behavior. Close to $\varepsilon _d=0$, $S\approx 0$ over a finite range indicates maximal Andreev reflection (see Fig. 5 b), whereas a steeper slope of *S* ($|\frac{dS}{d\varepsilon _d}|$) signals minimal Andreev and dominant quasiparticle transport.

The thermal conductance $\kappa$ (Fig. 6c) follows a trend similar to that of *G*, with no significant change in shape. The qualitatively distinct behavior of $\kappa$ for the two configurations suggests that $\kappa$ can serve as a potential probe for identifying gap anisotropy in quantum dot-based hybrids, as also demonstrated in some experiments under finite thermal bias and/or magnetic field^50,93,94^. Importantly, this distinction appears without any change in the temperature ($T_{TSC}$). The *ZT* (Fig. 6d) maximum amplitude is achieved at almost the same $|\varepsilon _d| \approx 0.6\Delta _0$ regardless of $\beta$. The magnitude of *ZT* does not differ significantly between the parallel and perpendicular cases. Any orientation of the TSC axis of symmetry in between these two orientations would offer the comparable range of ZT. Thus thermoelectric efficiency, quantified by *ZT*, remains almost independent of QD-TSC orientation, although the qualitative differences in the $G, S, \kappa$ can be significant.

### ABM state-parallel configuration

**Figure 7:**
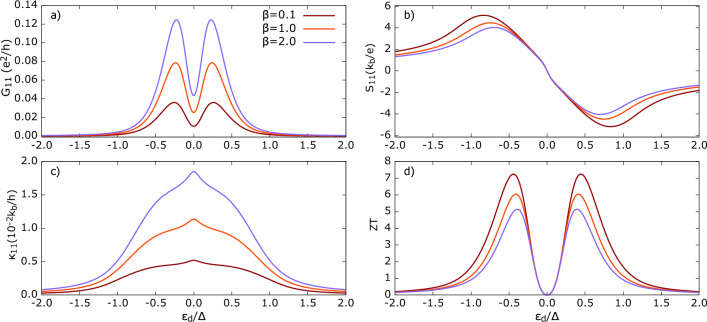
Thermoelectric coefficients for ABM state in *parallel* configuration. (**a**) electrical conductance (**b**) thermopower (**c**) thermal conductance (**d**) figure of merit. The other parameters: U=0, $\Gamma _{FM}=\Gamma _{TSC}=k_bT=0.1\Delta_0$, $p=0.5$. $\beta$ is the parameter of the Gaussian weight, centered around $\theta =0$.

The ABM state has a gap $\Delta _{ABM}=\Delta _{0}\sin {\theta }\exp {(i\phi )}$, which reaches its maximum in the equatorial plane of the momentum space of the superconductor. Therefore, the gap nodes of the state exist at the exact poles ($\theta =0, \pi )$ , where $\theta$ is measured from the symmetry axis of the SC gap, and hence two point nodes exist. In the parallel configuration for the ABM state, the polar axis, being the axis of symmetry of the SC gap, is parallel to the axis of tunneling, and the angular weighting is naturally centered around $\theta =0$. As $\beta$ increases, the conductance (Fig 7a) reveals an increasing trend in the maxima and deepening of the valley between the two resonances. Prima facie, the shape and trend of these curves resemble those of the Polar state in perpendicular configuration (see Fig. 6). However, a distinguishing feature of the ABM state is that the ratio $G_{peak}/G_{valley(\varepsilon _d=0)}$ increases with $\beta$, whereas in the Polar state, it remains almost constant. The origin of this can be found in the tunneling coefficients: $T^{QP}$ (shown in the Supplementary information) for the ABM state exhibits a shape change with $\beta$ (while for the Polar state, it maintains both its shape and relative magnitude across the range of $|\varepsilon |\lesssim 0.5\Delta_0$ as $\beta$ is varied. Importantly, for the ABM state in parallel configuration in the defined quantum dot based hybrid, the total conductance is determined purely by quasiparticle tunneling, i.e. $G = G_{QP}$, and $G_{AR}=0$. Remarkably, the origin of the suppression of Andreev reflection is fundamentally different from the Polar-perpendicular case and is embedded in the phase component, $\exp {(i\phi )}$, of the TSC gap $\Delta (k)$. The $\theta$-dependence of the anomalous self-energy enters via the factor $\sin ^2{\theta } \exp {(-\beta \theta ^2)}$ in the numerator of the integrand and gives a finite contribution to $\Sigma ^{r}_{14}$. The denominator is not explicitly written, as it is the same for diagonal and non-diagonal components of the self-energy and is an even function of $\phi ,\theta$ and $\varepsilon$; therefore, it does not effect the symmetry based cancellation discussed further. The absence of Andreev tunneling is solely due to the vanishing of $\Sigma ^{r}_{14}$, connected with integration over azimuthal angle $\phi$, although $\theta$ integral is finite. As in the parallel configuration, the Gaussian weight doesn’t depend on the azimuthal angle, one has $\Sigma ^{r}_{ND} \propto \int _{-\pi }^{\pi } e^{i\phi } = 0$. This is a direct manifestation of destructive interference arising from the $\pi$*-phase shift*, that is the $\phi$-dependent contribution to the self-energy at $\phi$ and $\phi +\pi$ is exactly out of phase and therefore cancels. This effect in a quantum dot based hybrid has resulted in a nullified (triplet) Andreev reflection. Other signatures of this phenomenon can be referred to some exemplary articles^95–97^. The quasiparticle contributed conductance $G_{QP}$ itself varies in the range of $10^{-2}-10^{-1}$. If compared to the Polar-perpendicular case, it is an order of magnitude smaller.

This stark difference can also be explained by considering the density of states of Polar and ABM phase of TSC. The low energy DoS for the Polar state varies as, $\rho _{Pol}(\varepsilon )\propto |\varepsilon |$, whereas that for ABM state, $\rho _{ABM}(\varepsilon )\propto \varepsilon ^2$. Thus, the Polar state provides more low-energy quasiparticle states available for transport than the ABM state. A line node supplies a much larger phase space for low-energy excitations, since an entire circle on the Fermi surface is gapless. In contrast, the phase space associated with a point node is far smaller: only a narrow solid angle around each node contributes to low-energy ABM quasiparticles. As a result, quasiparticle transport is significantly reduced in the ABM state.

The thermopower shown in Fig. 7 b) reveals a similar $\beta$-dependence as the corresponding *S* obtained for the Polar state in the perpendicular configuration. Likewise, the thermal conductance $\kappa$ (Fig. 7c) exhibits significant broadening and a change in the overall shape. The heat conductance follows similar behavior as those for Polar-perpendicular case, with the exception that now, as $\beta$ grows from 0.1 to 2.0, $\kappa$ shows substantial growth, while the full width at half maximum decreases from $\approx$ 12% to 7% at each step. The temperature and coupling parameters are kept constant. Therefore, here, a relatively stronger growth in $\kappa$ amplitude compared to the variation in resonance width indicates that increasing $\beta$ predominantly brings efficient renormalization of energy transfer taking place through heat conductance, rather than substantially inducing a broadening mechanism. These observations reaffirm the sensitivity of $\kappa$ profile and magnitude to the anisotropic gap structure of the superconductor. Furthermore, a nonzero slope of *S* (Fig. 7 b) close to particle hole symmetry point confirms the absence of the Andreev reflection. Both |*S*| and *ZT* (Fig. 7d) are almost monotonously decreasing functions with respect to $\beta$, with maxima at approximately the same QD energy.

### ABM state-perpendicular configuration

**Figure 8:**
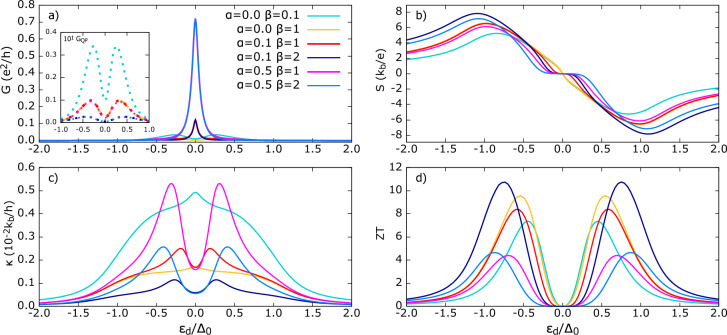
Thermoelectric coefficients for ABM state in *perpendicular* configuration. (**a**) electrical conductance (**b**) thermopower (**c**) thermal conductance (**d**) figure of merit. The inset in (**a**) shows only the quasiparticle contribution to conductance. The other parameters are: U=0, $\Gamma _{FM}=\Gamma _{TSC}=k_bT=0.1\Delta_0$, $p=0.5$.

In this configuration, QD is positioned in the equatorial plane of the momentum space of TSC crystal structure. The parameter $\alpha$ controls the range in $\phi$ over which the coupling strength of superconducting states is significant. The larger the $\alpha$, the narrower is this range around $\phi =0$. As a result, this $\phi$-dependent weighting breaks the symmetry of contributions coming from a cross-section of $|\Delta _{k}|$ for a given angle $\theta$, although the gap function itself is symmetric. We assume that $\alpha$ and $\beta$ are mutually independent. Due to the explicit $\phi$ dependence of $\Delta _{ABM}$, the transport coefficients vary with $\alpha$ as shown in Fig. 8. A strong central peak in conductance (Fig. 8 a) is associated with a dominant contribution of the Andreev reflection. We have $\Sigma ^{r}_{14} \propto \int _{-\pi }^{\pi } \exp {(i\phi )}\exp {(-\alpha \phi ^2)} d\phi =\int \cos {\phi }\exp {(-\alpha \phi ^2)}d\phi + i\int \sin {\phi }\exp {(-\alpha \phi ^2)}d\phi$. This clearly shows that for $\alpha = 0$, the integration vanishes. An increase in $\alpha$ rapidly decreases the integration contribution for $|\phi |>\pi /2$, where the function has a negative contribution to the self-energy. Consequently, over the full domain of $\phi$, i.e. $[-\pi ,\pi ]$, the total $\Sigma ^r_{14}$ (see self-energy Fig. S7 in the Supplementary information) increases. A finite $\alpha$ brings about the disruption of the perfect $\pi$ phase ($\phi$) antisymmetry, hence leading to finite Andreev reflection. Recall that for the ABM state with $\alpha = 0$, the Andreev conductance vanishes. This is a distinct feature found in the parallel configuration of ABM state. This can be understood as electrons in the SC condensate with momentum projection in the range $|\phi | > \pi /2$ are less and less likely to participate in the conductance (charge or heat). The individual contributions show that, as $\beta$ increases, $G_{QP}$ decreases (see inset), while $G_{AR}$ remains constant (corresponding to the central resonance peaks in panel a). In contrast, as $\alpha$ increases, $G_{QP}$ remains constant and $G_{AR}$ increases. In both cases, $G_{AR}$ dominates by one to two orders of magnitude. This behavior can be understood by examining the non-trivial structure of the Green’s function component responsible for the Andreev reflections, as $G_{AR} \propto T^{A} \propto \frac{|\Sigma ^r_{14(23)}|}{A-({\Sigma }^{r}_{14(23)})^2}$, where $A\equiv A(\varepsilon , \varepsilon _d, \Sigma _{ii}^{r})$. As shown in Fig. S7 in the Supplementary information, in the range $-0.5<\varepsilon/\Delta_0 <0.5$, when $\beta$ is kept constant, the diagonal term $\Sigma _{ii}^{r}$ remains insensitive to $\alpha$ variation. Furthermore, $\Sigma _{ii}^{r}$ and $\Sigma ^r_{14(23)}$ have comparable magnitudes. $|\Sigma ^r_{14}|$ shows enhancement with $\alpha$, thus increasing the numerator and simultaneously reducing the denominator, $A-({\Sigma }^{r}_{14(23)})^2$, consequently increasing the transmission probability of AR. This observation is experimentally relevant, as it suggests that an applied strain that modifies the Cooper pair amplitude in polar or azimuthal direction can independently change the Andreev and quasiparticle current.

The behavior of *S* can be understood from the interplay of the quasiparticle transport and Andreev reflection, similar to the discussion for the polar-parallel configuration. Beyond the subgap region, $\frac{dS}{d\varepsilon _d}$ remains nearly constant for a fixed $\beta$ when $\alpha$ is varied. Increasing $\beta$ shifts the thermally activated diffusion of charged particles toward energy levels further from the superconductor’s chemical potential, as is evident from the displacement of the maxima in *S*.

The behavior of thermal conductance $\kappa$ (Fig. 8c) is especially relevant parameter in the ABM-perpendicular case to understand the interplay of Andreev and quasiparticle dynamics. The overall $\kappa$ is reduced by an order of magnitude compared to the Polar state. In linear response, we have $\kappa \propto (L_{22}- \frac{L_{12}^2}{L_{11}})$, which measures heat conductance, provided there is no particle current. The coefficient $L_{22}$, is a proportionality factor between total thermal current (by effective particle or bare energy flow) and temperature difference between the two leads. This is in contrast to parallel configuration. The change of $\alpha$ has little to no effect on $L_{22}$. Since $L_{22} \propto (\varepsilon -\mu )T^{QP}$, (with $\mu =0$), electrons and holes with energy $\pm \varepsilon$ contribute equally to the heat current at $\varepsilon _d=0$. In the tunneling amplitude $T^{QP}({\varepsilon _d=0})$ (see Fig. S5 in the Supplementary information), we observe that near $\varepsilon =0$, $T^{QP}$ is the highest for the smallest value of $\beta$. This suggests that electrons and holes have higher and nearly equal tunneling probability. Their heat conductance contributions adds constructively, leading to a stronger bipolar effect and a pronounced $\kappa$ maximum at $\varepsilon _d = 0$ for $\alpha =0,\beta =0.1$. For higher $\beta$, the tunneling amplitude broadens, hence weakening the bipolar effect.

When $|\varepsilon _d| > 0.2\Delta_0$, the Andreev reflection still plays a significant role in $\kappa$ due to the term $-L_{12}^2/(G_{QP}+G_{AR})$ in $\kappa$. Increasing $\alpha$ (that is tighter azimuthal weighting) at a fixed $\beta$ brings about a substantial increment in $\kappa$ around $\varepsilon _d \approx 0.5$, consistent with the enhanced Andreev charge conductance. Therefore, the net effect of $\alpha$ and $\beta$ leads to qualitatively rich and tunable trends in $\kappa$ as a function of QD energy level. The relation between *ZT* maxima and $G^{AR}$ can be understood similar to the discussion in the Polar-parallel configuration. The *ZT* (Fig. 8d) attains its maximum for larger $\beta$ and minimum $\alpha$ throughout the range of $\varepsilon _d$ (maximum value achieved here is $\approx 12$). This shows that for the ABM state, maximizing the Cooper pair amplitude in the polar plane and suppressing it in the equatorial plane leads to maximum thermoelectric efficiency.

## Summary

We have analyzed the thermoelectric transport properties of a quantum dot-based hybrid structure where one lead is a *p*-wave, spin-triplet superconductor in the Polar or ABM phase, both exhibiting highly anisotropic gaps and having finite subgap density of states. We developed a phenomenological model to incorporate the anisotropy in the quantum dot-superconductor lead’s coupling, studying parallel and perpendicular configurations. We present thermoelectric transport coefficients within the linear response regime, using the non-equilibrium Green’s function method. Their dependence on the strength of anisotropy weighting parameters was evaluated. A benchmark comparison to the *s*-wave superconductor highlights the importance of anisotropy consideration in the theoretical analysis; otherwise, the Andreev contribution does not emerge and can lead to misleading conclusions. Our results reveal remarkable qualitative differences when the anisotropic coupling is introduced. The net Andreev reflection is completely suppressed for the Polar-perpendicular and ABM-parallel configurations, due respectively to polar angle dependence and intrinsic azimuthal $\pi$ phase shift.

By varying anisotropy strength and the orientation of the superconducting crystal, it is possible to maximize or minimize the triplet Andreev reflection compared to quasiparticle contribution. Thermopower exhibits finite subgap values and non-monotonic behavior, while thermal conductance shows great enhancement compared to the *s*-wave state. These results offer possible avenues for investigating a spin-triplet superconductor’s nodal structure.

Although the quantum dot is considered non-interacting, hence providing exact self-energies, the qualitative insights are robust. The modeling is sensitive to the design and geometry of the experimental devices. The formalism can be extended to consider non-equilibrium dynamics, the influence of magnetic field, and wide applicability to other superconducting states where the superconducting density of states is analytically accessible. Our work emphasizes the importance of anisotropy and nodal structure in controlling transport behavior in spin-triplet superconducting hybrid devices.

Implications for device design : i) Optimizing the spin-pure (triplet) transport and the role of ferromagnetic spin polarization: For finite triplet Andreev reflection (AR), fine-quality crystal structure is desirable, either a single crystal for an intrinsic TSC or constituent layers of a multi-layered engineered TSC system. Disorder hampers momentum selectivity for triplet AR. The gap-symmetry axis orientation, relative to the transport direction is a crucial parameter for measurement. A fabricated device has its natural $\alpha$ and $\beta$ equivalent parameter values, which can be varied by QD-TSC interface customization or by modifying the Fermi surface of the superconductor. For a fixed polarization *p*, the triplet AR (net from both spins’ orientations) is maximum for higher $\beta$ and $\alpha$ for Polar and ABM states, respectively. Therefore, narrowing the angular distribution of momentum boosts spin-pure AR. For a fixed Gaussian weight, a higher FM polarization is recommended to achieve greater spin-pure AR. This may decrease the total mixed-spin conductance, but enhance spin-resolved conductance.

ii) Optimizing the thermoelectric efficiency *ZT*: Comfortingly, from benchmarking results, the absolute value of *ZT* maxima is not drastically affected by the moderately disordered crystal. Our results show that AR enhances electrical conductance but is detrimental to *ZT* for a fixed orientation. The QD energy should be tuned (by gate voltage) outside of the high AR region. Substantially, we find that optimizing spin-pure triplet AR and *ZT* can be in conflict with each other. Therefore, the goal of device operation should be considered a priori. Among the chosen phases, ABM is more efficient than Polar.

## Additional information

Correspondence and requests for materials should be addressed to V.S.

## Supplementary Information

Supplementary Information 1.

## Data Availability

The datasets used and/or analyzed during the current study can be available from the corresponding author on reasonable request.
