# Supplementary material for: Impact of gap anisotropy of Polar and Anderson-Brinkman-Morel p-wave superconductors on thermoelectric properties of quantum dot hybrids
Source: Sci Rep. 2026 Apr 16;16:13629. doi: 10.1038/s41598-026-46160-2 (PMC13125262; doi:10.1038/s41598-026-46160-2)
Supplement: Supplementary file 1 — Supplementary Information 1. [file 41598_2026_46160_MOESM1_ESM.pdf]

Impact of gap anisotropy of Polar and Anderson-Brinkman-Morel p-wave superconductors on thermoelectric properties of quantum dot hybrids

Vrishali Sonar[1,*] **and Piotr Trocha**[1]

[1]Institute of Spintronics and Quantum Information, Faculty of Physics and Astronomy, Adam Mickiewicz University, Poznań, 61-614, Poland
[*]sonarvrishali@gmail.com, vrison@amu.edu.pl

Green's function

The retarded Green's function G^r , represented as a 4×4 matrix in Nambu space using Zubarev notation, is given by:

$$\mathbf{G}^r = \left\langle \left\langle \begin{pmatrix} d_\uparrow & d_\downarrow^\dagger & d_\downarrow & d_\uparrow^\dagger \end{pmatrix}^T \mid \begin{pmatrix} d_\uparrow^\dagger & d_\downarrow & d_\downarrow^\dagger & d_\uparrow \end{pmatrix} \right\rangle \right\rangle . \tag{1}$$

We derive $\mathbf{G}^r$ using the equation-of-motion method. Given that the Coulomb interaction within the quantum dot is set to zero ($U = 0$), the Green's functions are exact. By applying equation-of-motion (EOM),

$$\varepsilon \langle\langle A : B \rangle\rangle = \langle \{A, B\} \rangle + \langle\langle [A, H] : B \rangle\rangle \tag{2}$$

to the elements of $\mathbf{G}^r$, we obtain following equations for the components of Green's function,

$$(\varepsilon - \varepsilon_{d\sigma} - \Sigma_{N\sigma}^{(0)e}) \langle\langle d_\sigma | d_{\sigma'}^\dagger \rangle\rangle = \delta_{\sigma\sigma'} + U \langle\langle d_\sigma n_{\bar{\sigma}} | d_{\sigma'}^\dagger \rangle\rangle + V_{k\sigma}^{TSC} \sum_k \langle\langle b_{k\sigma} | d_{\sigma'}^\dagger \rangle\rangle , \tag{3}$$

$$(\varepsilon - \varepsilon_{d\sigma} - \Sigma_{N\sigma}^{(0)e}) \langle\langle d_\sigma | d_{\sigma'} \rangle\rangle = U \langle\langle d_\sigma n_{\bar{\sigma}} | d_{\sigma'} \rangle\rangle + V_{k\sigma}^{TSC} \sum_k \langle\langle b_{k\sigma} | d_{\sigma'} \rangle\rangle . \tag{4}$$

Similarly,

$$(\varepsilon + \varepsilon_{d\sigma} - \Sigma_{N\sigma}^{(0)h}) \langle\langle d_\sigma^\dagger | d_{\sigma'}^\dagger \rangle\rangle = -U \langle\langle d_\sigma^\dagger n_{\bar{\sigma}} | d_{\sigma'}^\dagger \rangle\rangle - V_{k\sigma}^{TSC*} \sum_k \langle\langle b_{-k\sigma}^\dagger | d_{\sigma'}^\dagger \rangle\rangle , \tag{5}$$

$$(\varepsilon + \varepsilon_{d\sigma} - \Sigma_{N\sigma}^{(0)h}) \langle\langle d_\sigma^\dagger | d_{\sigma'} \rangle\rangle = \delta_{\sigma\sigma'} - U \langle\langle d_\sigma^\dagger n_{\bar{\sigma}} | d_{\sigma'} \rangle\rangle - V_{k\sigma}^{TSC*} \sum_k \langle\langle b_{-k\sigma}^\dagger | d_{\sigma'} \rangle\rangle . \tag{6}$$

Here $\Sigma_{N\sigma}^{(0)e(h)}$ denotes the electron (hole) component of the non-interacting self-energy due ferromagnetic lead-quantum dot coupling. On the RHS, we made assumption of spin-momentum-independent tunneling amplitudes i.e. $V_{k\sigma}^{TSC} = V^{TSC}$.

The triplet superconductor gap function can be represented as a 2×2 complex matrix in spin space as

$$\mathbf{\Delta}(k) = \begin{bmatrix} \Delta_{\sigma\sigma}^k & \Delta_{\sigma\bar{\sigma}}^k \\ \Delta_{\bar{\sigma}\sigma}^k & \Delta_{\bar{\sigma}\bar{\sigma}}^k \end{bmatrix} \tag{7}$$

with $\mathbf{\Delta}(k)$ obeying the relation $\mathbf{\Delta}(k) = -\mathbf{\Delta}(-k)^T$ i.e. $\mathbf{\Delta}(k)_{\sigma_1\sigma_2} = -\mathbf{\Delta}(-k)_{\sigma_2\sigma_1}$.

Applying the EOM to $\langle\langle b^{(\dagger)}_{k\sigma}|d^{(\dagger)}_{\sigma'}\rangle\rangle$ gives,

$$(\varepsilon-\varepsilon_{ks\sigma})A-(\Delta^{k}_{\sigma\sigma}-\Delta^{-k}_{\sigma\sigma})C-(\Delta^{k}_{\sigma\bar{\sigma}}-\Delta^{-k}_{\bar{\sigma}\sigma})D=V_{s\sigma}\langle\langle d_{\sigma}|d^{\dagger}_{\sigma'}\rangle\rangle, \tag{8a}$$

$$(\varepsilon-\varepsilon_{ks\bar{\sigma}})B-(\Delta^{k}_{\bar{\sigma}\sigma}-\Delta^{-k}_{\sigma\bar{\sigma}})C-(\Delta^{k}_{\bar{\sigma}\bar{\sigma}}-\Delta^{-k}_{\bar{\sigma}\bar{\sigma}})D=V_{s\bar{\sigma}}\langle\langle d_{\bar{\sigma}}|d^{\dagger}_{\sigma'}\rangle\rangle, \tag{8b}$$

$$-(\Delta^{k*}_{\sigma\sigma}-\Delta^{-k*}_{\sigma\sigma})A-(\Delta^{k*}_{\sigma\bar{\sigma}}-\Delta^{-k*}_{\bar{\sigma}\sigma})B+(\varepsilon+\varepsilon_{-ks\sigma})C=-V_{s\sigma}\langle\langle d^{\dagger}_{\sigma}|d^{\dagger}_{\sigma'}\rangle\rangle, \tag{8c}$$

$$-(\Delta^{k*}_{\bar{\sigma}\sigma}-\Delta^{-k*}_{\sigma\bar{\sigma}})A-(\Delta^{k*}_{\bar{\sigma}\bar{\sigma}}-\Delta^{-k*}_{\bar{\sigma}\bar{\sigma}})B+(\varepsilon+\varepsilon_{-ks\bar{\sigma}})D=-V_{s\bar{\sigma}}\langle\langle d^{\dagger}_{\bar{\sigma}}|d^{\dagger}_{\sigma'}\rangle\rangle. \tag{8d}$$

Here, $A=\langle\langle b_{k\sigma}|d^{\dagger}_{\sigma'}\rangle\rangle$, $B=\langle\langle b_{k\bar{\sigma}}|d^{\dagger}_{\sigma'}\rangle\rangle$, $C=\langle\langle b^{\dagger}_{-k\sigma}|d^{\dagger}_{\sigma'}\rangle\rangle$, $D=\langle\langle b^{\dagger}_{-k\bar{\sigma}}|d^{\dagger}_{\sigma'}\rangle\rangle$. By solving the coupled equations (8), four variables A, B, C, D can be obtained in terms of the elements of the reduced Green's functions given by Eq. (1). For the Polar and ABM state phases discussed in the main text, $\Delta_{\sigma\bar{\sigma}}=0=\Delta_{\bar{\sigma}\sigma}$. we assume QD energy level $\varepsilon_{d\sigma}$ to be spin independent i.e. $\varepsilon_{d\sigma}=\varepsilon_{d}$. This significantly simplifies the coupled system of Eq. (8), resulting in the following analytical form of the dot's Green function:

$$G^{r}_{11}=\frac{1}{\varepsilon-\varepsilon_{d\sigma}-\Sigma^{(0)e}_{N\sigma}-\Sigma^{r}_{11,TSC}+\Sigma^{r}_{14,TSC}P}\quad \text{with } P=\frac{\Sigma^{r*}_{14,TSC}}{\varepsilon+\varepsilon_{d\sigma}-\Sigma^{(0)h}_{N\sigma}-\Sigma^{r}_{14,TSC}} \tag{9a}$$

$$G^{r}_{41}=P\,G^{r}_{11} \tag{9b}$$

$$G^{r}_{12}=0 \tag{9c}$$

$$G^{r}_{13}=0 \tag{9d}$$

We further present the analytical expression for superconductor self-energy in Polar and ABM state.

Self-energy

The general form of self-energy elements due to QD -TSC coupling in the Nambu space (see Eq. (1)) for the Polar or ABM state is given as,

$$\Sigma^{r}_{11,TSC}=\sum_{k}|V^{TSC}_{k\sigma}|^{2}\frac{\varepsilon+\varepsilon_{k\sigma}}{(\varepsilon+\varepsilon_{k\sigma})(\varepsilon-\varepsilon_{k\sigma})-\Delta^{2}_{k}} \tag{10a}$$

$$\Sigma^{r}_{14,TSC}=\sum_{k}|V^{TSC}_{k\sigma}|^{2}\frac{\Delta_{k}}{(\varepsilon+\varepsilon_{k\sigma})(\varepsilon-\varepsilon_{k\sigma})-\Delta^{2}_{k}} \tag{10b}$$

(10c)

A useful identity for converting the momentum sum into an integral is,

$$\sum_{k}\to V\int\frac{d^{3}k}{(2\pi)^{3}}=\int\frac{d\Omega_{k}}{4\pi}\int d\varepsilon_{k}N(\varepsilon_{k})\approx\frac{N_{0}}{4\pi}\int_{0}^{\pi}\sin\theta\,d\theta\int_{-\pi}^{\pi}d\phi\int_{-D}^{D}d\varepsilon_{k} \tag{11}$$

where $N(\varepsilon_{k})\approx N(\varepsilon_{F})=N_{0}$ is the density of states of normal electrons at the Fermi level, $d\Omega_{k}=\sin\theta d\theta d\phi$ denotes the solid angle subtended by the momentum vector in the momentum space of superconducting electrons and D represents the energy band width. Further, in the derivation of self-energy, we assume, $\varepsilon_{k\sigma}=\varepsilon_{k\bar{\sigma}}=\varepsilon_{-k\sigma}$ and place $D\to\infty$. We further show the *bare* self-energy, i.e., the self-energy obtained in the absence of spatial weighting of the coupling matrix elements between QD and TSC.

Self-energy due to superconductor in Polar state

Gap function for Polar state is $\Delta_{Pol} = \Delta_0 \cos\theta$. From equation (10), the integration over momentum in polar coordinates leads to

$$\Sigma^r_{Pol,11} = -\frac{i\Gamma_{TSC}}{2}\frac{\varepsilon}{\Delta_0}\left[\arcsin\left(\frac{\Delta_0}{\varepsilon}\right)\Theta(|\varepsilon|-\Delta_0) + \left(-i\ln\frac{\Delta_0+\sqrt{\Delta_0^2-\varepsilon^2}}{|\varepsilon|} + sign(\varepsilon)\frac{\pi}{2}\right)\Theta(\Delta_0-|\varepsilon|)\right], \tag{12a}$$

$$\Sigma^r_{Pol,14} = 0. \tag{12b}$$

Here, $\Theta(x)$ represents the Heaviside function and $\Gamma_{TSC} = 2\pi N_0|V^{TSC}|^2$. The vanishing of $\Sigma^r_{Pol,14}$ results due to effective integrand being an odd function of θ. Therefore, in the absence of weighting, Polar self-energy matrix is always diagonal. When weighting is introduced (as discussed in the main text), the non-diagonal (Σ^r_{14}) elements becomes finite.

Self-energy due to superconductor in ABM state

Gap function for ABM state is $\Delta_{ABM} = \Delta_0 \sin\theta \exp(i\phi)$. In this case the self-energy acquires the form,

$$\begin{aligned}
\Sigma^r_{ABM,11} &= \frac{-i\Gamma_{TSC}}{4}\frac{\varepsilon}{\Delta_0}\ln\left|\frac{\varepsilon+\Delta_0}{\varepsilon-\Delta_0}\right| \\
&= \frac{-i\Gamma_{TSC}}{4}\frac{\varepsilon}{\Delta_0}\left[\ln\left(\frac{\varepsilon+\Delta_0}{\varepsilon-\Delta_0}\right)\Theta(|\varepsilon|-\Delta_0) + \ln\left(\frac{\varepsilon+\Delta_0}{\Delta_0-\varepsilon}\right)\Theta(\Delta_0-|\varepsilon|) - i\pi\Theta(\Delta_0-|\varepsilon|)\right] \\
\Sigma^r_{ABM,14} &= (\frac{iN_0|V_s|^2}{2})\frac{\Delta_0}{\sqrt{\varepsilon^2-\Delta_0^2}}(-k)\int_0^{2\pi} d\phi e^{i\phi} I\left(\arcsin\frac{-1}{k},k\right)
\end{aligned} \tag{13}$$

with

$$I\left(\arcsin\frac{-1}{k},k\right) = \left[\int_0^{\arcsin\frac{-1}{k}} du\sqrt{1-k^2\sin^2 u}\right] \tag{14}$$

The parameters in $\Sigma^r_{ABM,14}$ are defined as $k = \frac{i}{a}$, $a = \frac{\Delta_0^2}{\varepsilon^2-\Delta_0^2}$, $\sin u = \tilde{x} = iax$. The integral $I\left(\arcsin\frac{-1}{k},k\right)$ represents an elliptic function, such that its characteristic parameter k can have a magnitude lesser or greater than unity and can be an imaginary number. Because the ABM pairing contains the azimuthal phase factor ($e^{i\phi}$), the angular integration $\int_0^{2\pi} e^{i\phi}\, d\phi = 0$. Therefore, the off-diagonal element of bare self-energy vanishes $\Sigma^r_{ABM,14} = 0$. A finite off-diagonal element appear only when the direction dependent weighting is included, as discussed in the main text.

Tunneling Coefficients

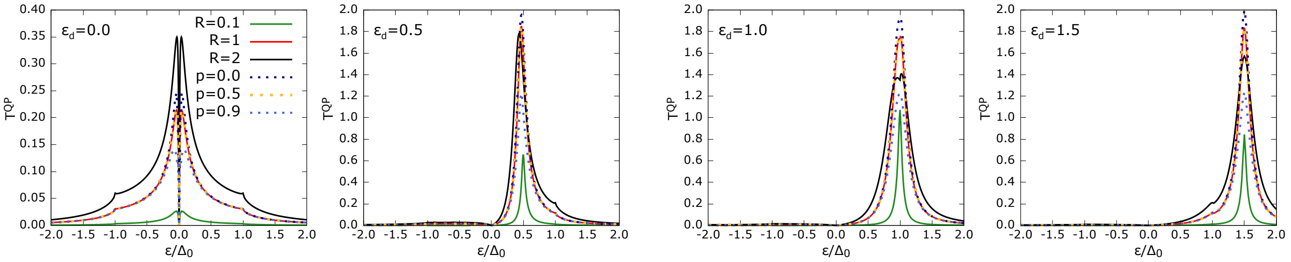

Figure S1. Tunneling coefficient (quasiparticle contribution) for the Polar state in the averaged self-energy case (without weighting), shown as a function of the tunneling electron energy ε. The Andreev reflection is absent, $T^A = 0$, due to the vanishing off-diagonal component of the self-energy i.e. $\Sigma^r_{14} = 0$. The solid lines represent variation of the QD–TSC coupling for a fixed polarization $p = 0.5$. The dotted lines indicate the variation of the FM lead polarization p for a fixed coupling $R = 1$. Other parameters are set to: $U = 0$, $\Gamma_{FM} = 0.1$, $\Gamma_{SC} = 0.1$, and $k_B T = 0.1$ in units of Δ_0

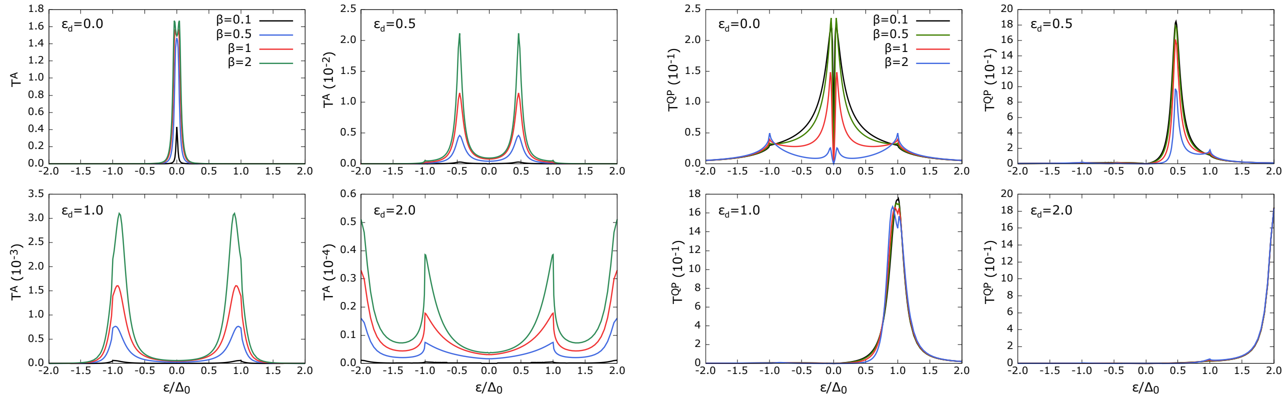

(a) (b)

Figure S2. Tunneling coefficients for the Polar state in *parallel* configuration: (a) Andreev reflection and (b) quasiparticle tunneling as a function of the tunneling electron's energy ε, for the indicated values of quantum-dot energy level ε_d. Other parameters are $U = 0$, $\Gamma_{FM} = 0.1$, $\Gamma_{SC} = 0.1$, $k_B T = 0.1$ in units of Δ_0 and $p = 0.5$.

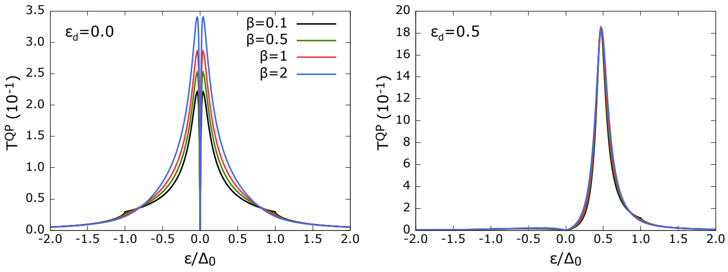

Figure S3. Tunneling coefficient for Polar state in *perpendicular* configuration as a function of energy ε of the tunneling electron. The Andreev tunneling coefficients are not shown, since $T^A = 0$. Other Parameters $U = 0$, $\Gamma_{FM} = 0.1$, $\Gamma_{SC} = 0.1$, $k_b T = 0.1$ in units of Δ_0 and $p = 0.5$.

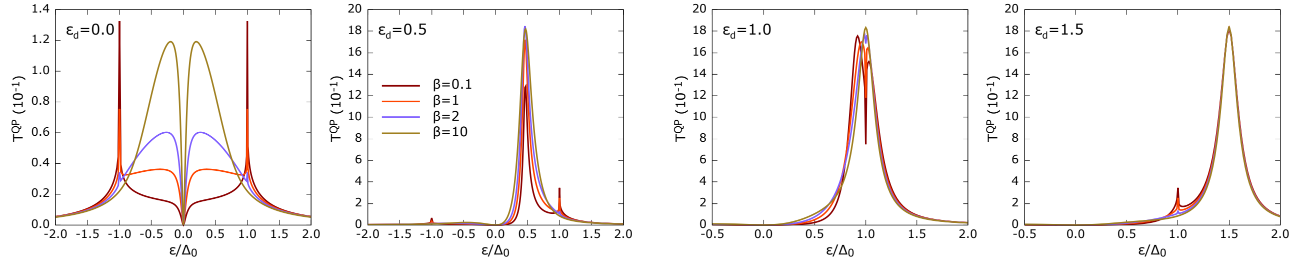

Figure S4. Tunneling coefficient for ABM state in *parallel* configuration as a function of energy ε of the tunneling electron. The Andreev tunneling coefficients are not shown, since $T^A = 0$. Parameters: $U = 0$, $\Gamma_{FM} = 0.1$, $\Gamma_{SC} = 0.1$, $k_b T = 0.1$ in units of Δ_0 and $p = 0.5$.

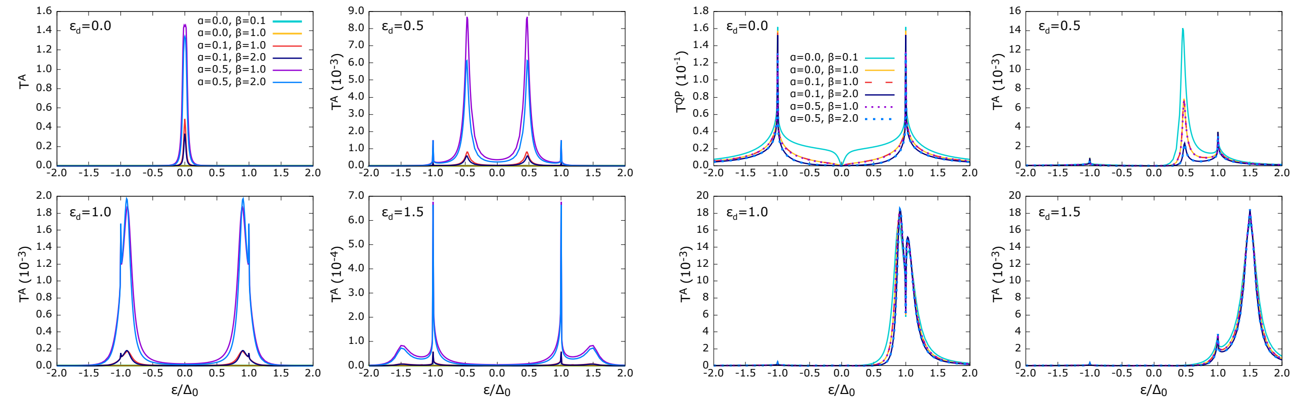

(a) (b)

Figure S5. Tunneling coefficients for ABM state in *perpendicular* configuration for a) Andreev b) Quasiparticle tunneling as a function of energy ε of the tunneling electron, for indicated values of quantum dot energy level ε_d. Other Parameters $U = 0$, $\Gamma_{FM} = 0.1$, $\Gamma_{SC} = 0.1$, $k_b T = 0.1$ in units of Δ_0 and $p = 0.5$.

Weighted Self-Energy

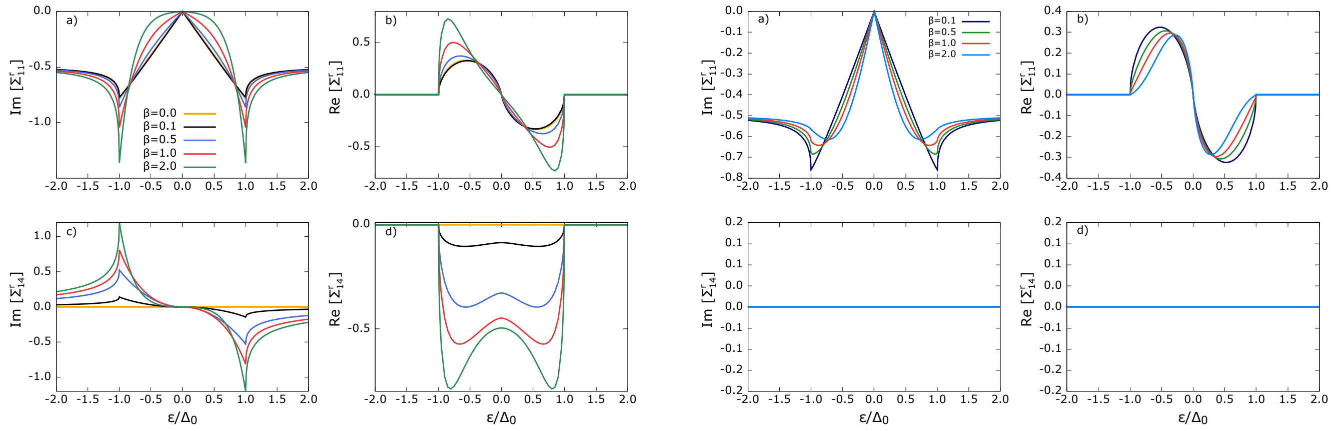

a) Parallel

b) Perpendicular

Figure S6. Self-energy for *Polar* state in a) Parallel b) Perpendicular configuration for given β parameters. The α parameters are not shown for the perpendicular case as the weight function cancels out with the normalization, therefore do not affect the self-energy. The 11th and 14th element of the self-energy in the Nambu space is shown. The finite spin-triplet Andreev reflection is associated with Σ^r_{14} being finite.

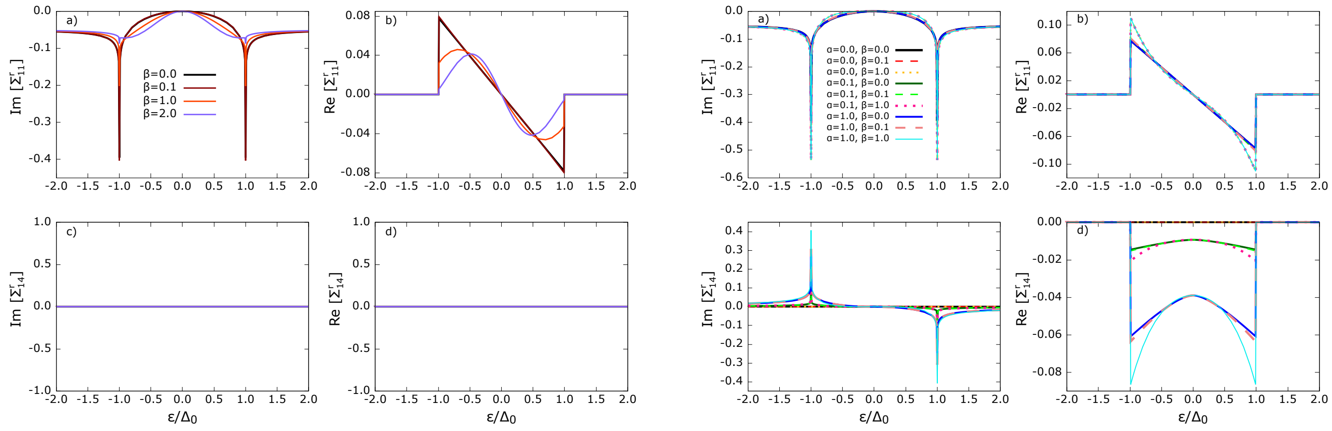

a) Parallel

b) Perpendicular

Figure S7. Self-energy for *ABM* state in a) Parallel b) Perpendicular configuration for given α, β parameters. The 11th and 14th element of the self-energy in the Nambu space is shown. The finite spin-triplet Andreev reflection is associated with Σ^r_{14} being finite.

Relation between position of ZT and Andreev reflection

.

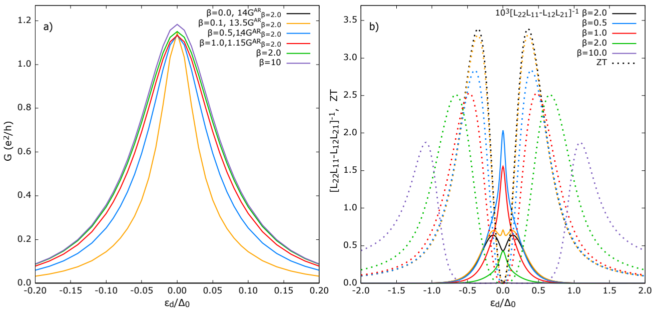

(a) Polar state-Parallel Configuration

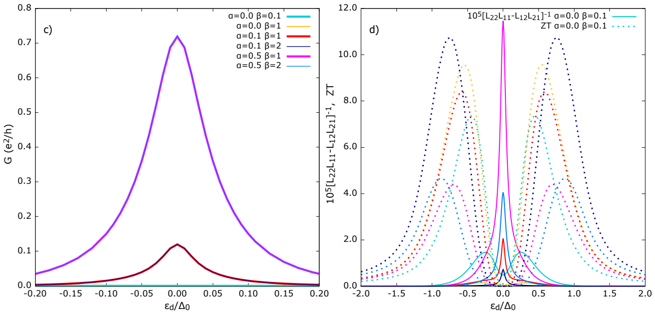

(b) ABM state-Perpendicular Configuration

Figure S8. a) Andreev reflection conductance (scaled to simplify comparison of resonance width distribution) and b) figure of merit ZT and denominator of ZT i.e. $[L_{22}L_{11} - L_{12}L_{21}]^{-1}$ for Polar state at the indicated values of the Gaussian weights. On x axis-Quantum dot's energy level ε_d/Δ_0. Similarly c) and d) for the ABM state.

Possible impact of finite intra-dot Coulomb correlations

A finite Coulomb interaction introduces an additional charging energy for double occupancy of the quantum dot, leading to a splitting of the spectral function and corresponding conductance resonances at ε_d and $\varepsilon_d + U$ due to the Coulomb blockade effect. In the regime $\Gamma \ll U$, the spectral function and conductance can exhibit two Hubbard-like resonances separated approximately by U (see e.g. Ref.[1] and Fig. S2 of its supplementary information and Ref.[2]).

Finite U can affect Andreev and quasiparticle transport differently. As U increases from zero to some finite value, the Andreev reflection resonance may split and its amplitude can change, while further variation of U primarily shifts the resonance positions without significantly altering their widths. For a certain parameter regimes, an additional Andreev reflection maximum may appear near $\varepsilon_d = -U/2$[3]. At sufficiently low temperatures and strong interactions ($U \gg \Gamma$ and $T < T_K$, where T_K is the Kondo temperature), an additional sharp resonance associated with the Kondo effect may appear within the Coulomb blockade region[4–6].

However, unlike conventional s-wave case, certain p-wave states (Polar and ABM considered here) have finite quasiparticle density of states in the subgap (measured with respect to the maximum gap Δ_0). Consequently, the split Andreev resonances may not be clearly distinguishable from quasiparticle resonances. The qualitative behavior of the split quasiparticle resonances will also depend on the absolute value of U, since U can shift the resonances inside or outside the $\pm\Delta_0$ region, where the quasiparticle density of states is finite and exhibits distinct functional forms.

The above discussion is qualitative and a more rigorous analysis would require solving the interacting Green's functions (e.g., using equation-of-motion techniques), which is beyond the scope of the present work. Finite U makes the system interacting and therefore not exactly solvable. Consequently, the self-energy needs to be approximated (the Hubbard-I approximation is sufficient to explain Coulomb blockade) and the dot occupation should then be computed self-consistently.

References

1. Sonar, V. & Trocha, P. Spin dependent thermoelectric transport in a multiterminal quantum dot hybrid including a superconductor and ferromagnets. *Sci. Reports* **15**, 14509, 10.1038/s41598-025-94991-2 (2025).

2. Verma, S. & Singh, A. Non-equilibrium thermoelectric transport across normal metal–quantum dot–superconductor hybrid system within the Coulomb blockade regime. *J. Physics: Condens. Matter* **34**, 155601, 10.1088/1361-648X/ac4ced (2022).

3. Trocha, P. & Barnaś, J. Spin-dependent thermoelectric phenomena in a quantum dot attached to ferromagnetic and superconducting electrodes. *Phys. Rev. B* **95**, 165439, 10.1103/PhysRevB.95.165439 (2017).

4. Martinek, J. *et al.* Kondo effect in quantum dots coupled to ferromagnetic leads. *Phys. Rev. Lett.* **91**, 127203, 10.1103/PhysRevLett.91.127203 (2003).

5. Świrkowicz, R., Wilczyński, M., Wawrzyniak, M. & Barnaś, J. Kondo effect in quantum dots coupled to ferromagnetic leads with noncollinear magnetizations. *Phys. Rev. B* **73**, 193312, 10.1103/PhysRevB.73.193312 (2006).

6. Georges, A. The beauty of impurities: Two revivals of Friedel's virtual bound-state concept. *Comptes Rendus Physique* **17**, 430–446, 10.1016/j.crhy.2015.12.005 (2016).
